# Interferon Regulatory Factor 5 Controls Necrotic Core Formation in Atherosclerotic Lesions by Impairing Efferocytosis

**DOI:** 10.1161/CIRCULATIONAHA.117.027844

**Published:** 2017-09-19

**Authors:** Anusha N. Seneviratne, Andreas Edsfeldt, Jennifer E. Cole, Christina Kassiteridi, Maarten Swart, Inhye Park, Patricia Green, Tariq Khoyratty, David Saliba, Michael E. Goddard, Stephen N. Sansom, Isabel Goncalves, Rob Krams, Irina A. Udalova, Claudia Monaco

**Affiliations:** From Kennedy Institute of Rheumatology, Nuffield Department of Orthopaedics, Rheumatology and Musculoskeletal Sciences, University of Oxford, United Kingdom (A.N.S., A.E., J.E.C., C.K., M.S., I.P., P.G., T.K., D.S., M.E.G., S.N.S., I.A.U., C.M.); Department of Bioengineering, Imperial College London, United Kingdom (A.N.S., R.K.); Experimental Cardiovascular Research Unit, Clinical Research Centre, Clinical Sciences Malmö, Lund University, Sweden (A.E., I.G.); Department of Cardiology, Skåne University Hospital, Lund/Malmö, Sweden (A.E., I.G.); and School of Engineering and Materials Science, Queen Mary University of London, United Kingdom (R.K.).

**Keywords:** atherosclerosis, CD11c, efferocytosis, IRF5, macrophages

## Abstract

**Background:**

Myeloid cells are central to atherosclerotic lesion development and vulnerable plaque formation. Impaired ability of arterial phagocytes to uptake apoptotic cells (efferocytosis) promotes lesion growth and establishment of a necrotic core. The transcription factor interferon regulatory factor (IRF)-5 is an important modulator of myeloid function and programming. We sought to investigate whether IRF5 affects the formation and phenotype of atherosclerotic lesions.

**Methods:**

We investigated the role of IRF5 in atherosclerosis in 2 complementary models. First, atherosclerotic lesion development in hyperlipidemic apolipoprotein E-deficient (ApoE^-/-^) mice and ApoE^-/-^ mice with a genetic deletion of IRF5 (ApoE^-/-^Irf5^-/-^) was compared and then lesion development was assessed in a model of shear stress-modulated vulnerable plaque formation.

**Results:**

Both lesion and necrotic core size were significantly reduced in ApoE^-/-^Irf5^-/-^ mice compared with IRF5-competent ApoE^-/-^ mice. Necrotic core size was also reduced in the model of shear stress-modulated vulnerable plaque formation. A significant loss of CD11c^+^ macrophages was evident in ApoE^-/-^Irf5^-/-^ mice in the aorta, draining lymph nodes, and bone marrow cell cultures, indicating that IRF5 maintains CD11c^+^ macrophages in atherosclerosis. Moreover, we revealed that the CD11c gene is a direct target of IRF5 in macrophages. In the absence of IRF5, CD11c^-^ macrophages displayed a significant increase in expression of the efferocytosis-regulating integrin-β3 and its ligand milk fat globule-epidermal growth factor 8 protein and enhanced efferocytosis in vitro and in situ.

**Conclusions:**

IRF5 is detrimental in atherosclerosis by promoting the maintenance of proinflammatory CD11c^+^ macrophages within lesions and controlling the expansion of the necrotic core by impairing efferocytosis.

## Abstract 2

Supplemental Digital Content is available in the text.

**Clinical Perspective**

**What Is New?**

- Using murine models of atherosclerosis, we provide evidence for a pathogenic role for the transcription factor interferon regulatory factor(IRF5) in atherosclerosis.
- Atherosclerosis-prone apolipoprotein E-deficient (ApoE^-/-^) mice deficient in IRF5 (ApoE^-/-^Irf5^-/-^) exhibit reduced atherosclerotic lesion and necrotic core formation compared with IRF5-competent ApoE^-/-^ mice.
- We reveal that development of the lesion necrotic core is controlled by IRF5 through impairment of macrophage efferocytosis (dead cell removal) through the molecules milk fat globule-epidermal growth factor 8 protein and integrin β-3.
- We further demonstrate that the CD11c gene is a direct target of IRF5 in macrophages and that IRF5 is important in maintaining CD11c^+^ macrophages in atherosclerotic lesions.

**What Are the Clinical Implications?**

- Larger necrotic cores are associated with an unstable atherosclerotic plaque phenotype, which is more likely to rupture and manifest as acute clinical complications.
- IRF5 contributes to a vulnerable plaque phenotype by maintaining proinflammatory CD11c^+^ macrophages in the plaque and impairing efferocytosis, causing larger necrotic cores.
- Currently, no therapies are available that specifically target plaque inflammation and efferocytosis and modulate plaque stability.
- IRF5 is a potential therapeutic target because IRF5 inhibition could reduce plaque inflammation and necrotic core size and therefore promote a stable plaque phenotype with lower risk of acute clinical complications.

Atherosclerosis, through causing heart attacks and strokes, is responsible for nearly half of deaths worldwide.^1^ The presence of mononuclear phagocytes within the innermost intimal layer of the arterial wall is the hallmark of atherosclerosis and is necessary for its development.^2,3^ The variety of macrophage subsets described in atherosclerosis is testament to the plasticity of this cell, with cell programming driven by lipids, inflammation, and hemorrhage.^4^ Macrophages are crucial for programmed apoptotic cell removal or efferocytosis during atherogenesis and inflammation.^5^ Impaired efferocytosis, resulting from either the persistent expression of don’t eat me signals such as CD47 on apoptotic cells^6^ or the cleavage of efferocytic receptors,^7^ leads to larger necrotic cores that are a feature of high-risk lesions. The mechanisms linking macrophage programming with efferocytosis function are ill defined.

The transcription factor interferon regulatory factor (IRF)-5 is a master regulator of inflammatory macrophage programming.^8,9^ Granulocyte macrophage colony-stimulating factor (GM-CSF) and interferon-γ treatment increases IRF5 expression.^8,9^ Toll like receptor activation of myeloid differentiation primary response gene 88 signaling leads to phosphorylation of IRF5 and its nuclear translocation.^9^ Genetic deletion of IRF5 protects from murine inflammatory arthritis and insulin resistance in diet-induced obesity,^10,11^ while its inhibition with nanoparticles decreases myocardial infarct size.^12^

Despite the widespread prevalence of atherosclerosis in the population, acute complications arise in vulnerable patients with unfavorable plaque morphologies, such as thin-cap fibroatheroma (TCFA). TCFA is characterized by a large necrotic core surrounded by a thin protective fibrous cap containing fewer smooth muscle cells.^13^ We have previously shown that hemodynamic factors may be relevant to TCFA formation. Low shear stress (LSS) occurs in the inner curvatures of vessels (eg, in the aortic arch and coronary arteries) and upstream of stenosis. In murine models, exposure to LSS leads to the development of plaque morphologies that resemble human TCFA.^14^ We have also previously shown that IRF5 expression is enhanced in arterial regions exposed to LSS.^15^

In the present study, we evaluated the effect of IRF5 deficiency on the development of atherosclerosis in 2 complementary murine models. Loss of IRF5 expression reduced aortic lesion and necrotic core size in atherosclerosis-prone apolipoprotein E-deficient (ApoE^-/-^) mice. Furthermore, arterial segments exposed to LSS developed smaller necrotic cores and displayed a higher content of smooth muscle cells in IRF5-deficient ApoE^-/-^ mice. Loss of IRF5 significantly reduced CD11c expression and infiltration of CD11c^+^ macrophages in the aorta and draining lymph nodes while promoting the ability of macrophages to perform efferocytosis by integrin β-3 (Itgb3) and milk fat globule-epidermal growth factor 8 protein (Mfge8). Our data indicate that IRF5 is a key regulator of efferocytosis and necrotic core formation in atherosclerosis.

## Methods

### Mice

ApoE^-/-^ mice on a C57BL/6 background were purchased from Charles River Laboratories and bred in-house. Irf5^-/-^ mice on a C57BL/6 background (from Professor Irina Udalova) were bred with ApoE^-/-^ mice to generate ApoE^-/-^Irf5^-/-^ mice and their littermates (referred to as ApoE^-/-^ mice thereafter). Experimental animals were negative for the Dock2 mutation.^16^ Animals were housed under specific pathogen-free conditions and studied according to UK Home Office regulations and institutional guidelines. Only male mice were used in this study.

### Murine Lesion Analysis and Staining

Aortic root and cast-induced carotid lesion sections were stained and quantified as described in the online-only Data Supplement Methods. In situ efferocytosis was assessed in cast-induced carotid lesion sections stained for apoptosis (TUNEL), DNA (7AAD), and CD68 as described in online-only Data Supplement Methods.

### Analysis of Aortas and Para-Aortic Lymph Nodes (PALNs)

Aortas and PALNs were harvested from 20-week-old ApoE^-/-^ and ApoE^-/-^Irf5^-/-^ mice. Single-cell suspensions were stained with antibodies and analyzed by flow cytometry or RNA was extracted and gene expression was analyzed using reverse transcription polymerase chain reaction, performed using Taqman assays as described in the online-only Data Supplement Methods.

### Bone Marrow Macrophage Culture and Functional Assays

Bone marrow macrophages were isolated from ApoE^-/-^ and ApoE^-/-^Irf5^-/-^ mice and cultured with GM-CSF as described in online-only Data Supplement Methods. Functional assays including apoptosis, foam cell, phagocytosis, and efferocytosis assays (including siRNA knockdown of Itgb3 and Mfge8) were then performed as described in the online-only Data Supplement Methods

### Chromatin Immunoprecipitation and Next-Generation Sequencing

The IRF5 chromatin immunoprecipitation sequencing analysis was performed as previously described (accession number: E-MTAB-2661)^17^ to determine the role of IRF5 as a regulator of CD11c expression.

### Human Carotid Plaques

Human carotid plaques from the Carotid Plaque Imaging Project biobank were analyzed as described in the online-only Data Supplement Methods. Informed consent was given by each patient, and the study was approved by the local ethical committee.

### Statistical Methods

Data were analyzed with GraphPad Prism (v6.0c) or the R WRS2 package. Normally distributed variables are expressed as mean±SEM, and non-normally distributed variables are expressed as median and interquartile range (IQR). For aortic root lesion area and immunohistochemical staining in ApoE^-/-^ and ApoE^-/-^Irf5^-/-^ mice, the significance of changes in the main effects (time, genotype, and their interaction) was assessed using a robust 2-way ANOVA. For each parameter of interest in these data, Bonferroni-corrected nonparametric (Mann-Whitney *U*) planned tests were performed to assess the effect of genotype at each time point. Data from cast-induced carotid lesions were similarly analyzed using a mixed robust 2-way ANOVA to account for the paired observations of low and oscillatory stress. We used Bonferroni-corrected nonparametric (Mann-Whitney U) planned tests to assess the effect of (1) genotype within stress type (unpaired test), and (ii) stress type within genotype (paired test). The use of nonparametric and robust ANOVA methods was motivated by observations of non-normality and unequal group variances. The significant findings from the planned tests are reported in the figures, and results of the ANOVA analyses are summarized in Tables I–V in the online-only Data Supplement. In the remainder of the article, data were analyzed with Student’s *t* test, Mann-Whitney *U* test, 1-way ANOVA, and Spearman’s or Pearson’s correlation coefficient as appropriate. The Benjamini-Hochberg method was used to adjust the *P* values for multiple testing as necessary.

## Results

### IRF5 Is Expressed in Atherosclerotic Lesions by Myeloid Cells

Expression of IRF5 was examined in aortic root lesions of ApoE^-/-^ mice 12, 20, and 27 weeks of age. Maximal IRF5 expression (6.3±2.5% of lesion area) was observed at 20 weeks of age (Figure IA in the online-only Data Supplement). Using confocal microscopy, IRF5 staining in the atherosclerotic lesion was observed in CD68- and CD11c-expressing cells. Nuclear translocation could also be observed (Figure IB and C in the online-only Data Supplement). Conversely, expression of α-smooth muscle actin in the intima of the lesion did not colocalize with IRF5 staining (Figure ID in the online-only Data Supplement).

### IRF5 Deficiency Reduces Atherosclerotic Lesion and Necrotic Core Size

ApoE^-/-^ and ApoE^-/-^Irf5^-/-^ mice were fed a chow diet and euthanized at 15, 20, or 27 weeks of age. No difference in serum cholesterol levels was observed between ApoE^-/-^ and ApoE^-/-^Irf5^-/-^ mice at any time point examined (Table VI in the online-only Data Supplement). ApoE^-/-^Irf5^-/-^ mice were heavier than ApoE^-/-^ mice at 15 weeks of age, in keeping with published studies (Table VI in the online-only Data Supplement).^10^

**Figure 1.**
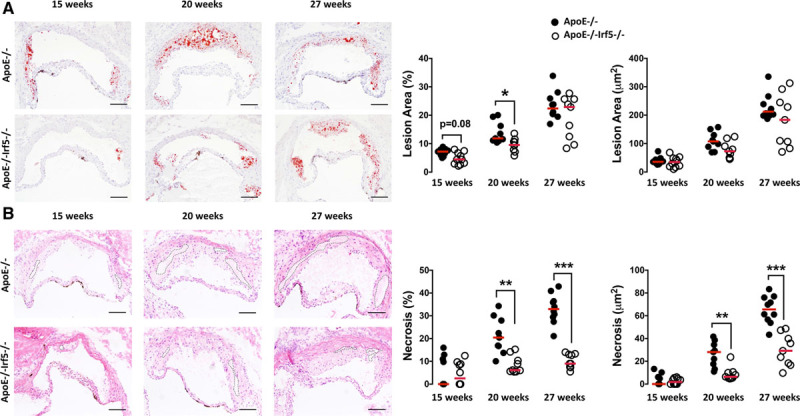
**IRF5 deficiency decreases lesion and necrotic core size in the aortic root of ApoE^-/-^ mice.** Representative images of aortic root sections from ApoE^-/-^ and ApoE^-/-^Irf5^-/-^ mice 15, 20, or 27 weeks of age stained with Oil Red O and hematoxylin for lesion area (**A**). Graphs show cross-sectional aortic root lesion area (x10^3^ μm^2^ and %) (**B**) hematoxylin and eosin for necrotic core delineation. Graphs show aortic root lesional necrotic core area (defined as anuclear, afibrotic, and eosin-negative areas) (x10^3^ μm^2^ and %). Dotted lines show necrotic core area. Each circle represents the mean area per individual mouse. Horizontal line denotes group median (n=9 to 10). Bars=100μm. ApoE indicates apolipoprotein E-deficient; and IRF, interferon regulatory factor. *P<0.05; **P<0.01; ***P<0.001.

Lesion size was assessed in ApoE^-/-^ and ApoE^-/-^Irf5^-/-^ mice at the level of the aortic root after staining with Oil Red O. ApoE^-/-^Irf5^-/-^ mice had smaller lesions than ApoE^-/-^ mice at 15 weeks of age (ApoE^-/-^ versus ApoE^-/-^Irf5^-/-^: 7.2% [IQR, 5.99–7.91] versus 4.44% [IQR, 2.99–6.48]; *P*=0.084). Similarly, IRF5-deficient ApoE^-/-^ mice had smaller lesions at 20 weeks of age (11.9% [IQR, 11.2–16.3] versus 9.5% [IQR, 7.85–10.7]; *P*=0.032; Figure 1A). No difference was observed at 27 weeks of age. The smaller lesion size was accompanied by a reduction in necrotic core formation, evaluated using hematoxylin and eosin staining according to established protocols.^18^ In contrast to ApoE^-/-^ mice, lesional necrotic core size was ≈70% smaller in ApoE^-/-^Irf5^-/-^ mice at 20 weeks of age (20.4% [IQR, 16.7–27.9] versus 6.14% [IQR, 5.63–10.3]; *P*=0.0023) and ≈73% smaller at 27 weeks of age (32.9% [IQR, 29.7–36.3] versus 9% [IQR, 7.5–13.1]; *P*=6.5x10^−5^; Figure 1B).

**Figure 2.**
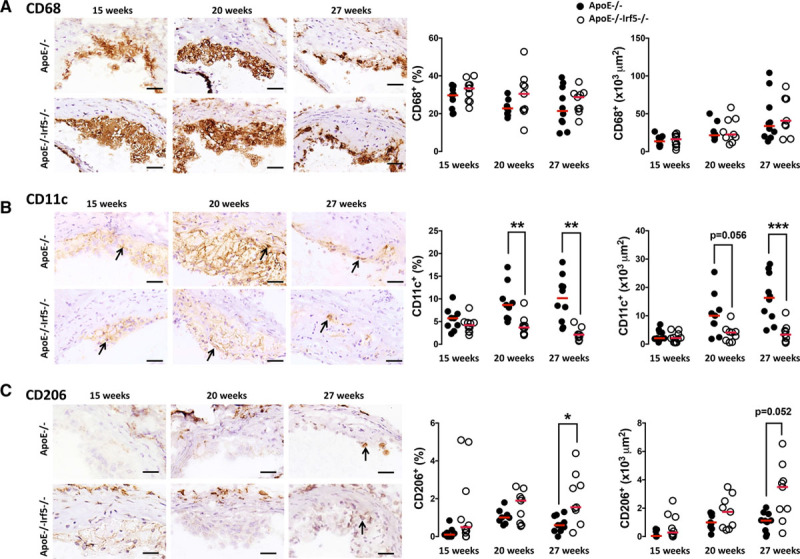
**IRF5 deficiency reduces CD11c expression in the aortic root.** **A**, Representative photomicrographs of aortic root sections from 15-, 20-, and 27-week-old ApoE^-/-^ and ApoE^-/-^Irf5^-/-^ mice stained with an antibody against CD68 (brown staining) and hematoxylin. Graphs show aortic root lesion area staining positive (x10^3^ μm^2^ and %) for CD68. **B**, Representative photomicrographs of aortic root sections from 15-, 20-, and 27-week-old ApoE^-/-^ and ApoE^-/-^Irf5^-/-^ mice stained with an antibody against CD11c (brown staining) and hematoxylin. Arrows highlight CD11c^+^ positive cells. Graphs show aortic root lesion area staining positive (x10^3^ μm^2^ and %) for CD11c. **C**, Representative photomicrographs of aortic root sections from 15-, 20-, and 27-week-old ApoE^-/-^ and ApoE^-/-^Irf5^-/-^ mice stained with an antibody against CD206 (brown staining) and hematoxylin. Graphs show aortic root lesion area staining positive (x10^3^ μm^2^ and %) for CD206 (n=9 to 10). Bars=100μm. Each circle represents the mean positive area per individual mouse. Horizontal line denotes group median. ApoE indicates apolipoprotein E-deficient; and IRF, interferon regulatory factor. *P<0.05; **P<0.01; ***P<0.001.

Lesions in the aortic root were further characterized by immunohistochemistry. A small increase in CD68 (Figure 2A) immunopositive area percentage was observed in the ApoE^-/-^Irf5^-/-^ mice across all the time points (*P*=0.045; Table II in the online-only Data Supplement). However, this difference did not achieve statistical significance in the planned tests conducted at the individual time points or when CD68 immunopositivity was expressed as absolute area.

CD11c^+^ myeloid cells contribute to lipid accumulation and the initiation of atherosclerosis.^19^ Hence, CD11c expression was examined. CD11c expression was significantly attenuated in aortic root lesions of ApoE^-/-^Irf5^-/-^ compared with ApoE^-/-^ mice with a 57% decrease at 20 weeks of age (8.65% [IQR, 5.8–10] versus 3.7% [IQR, 2.3–4.1]; *P*=0.0092) and an 80% reduction at 27 weeks of age (10.2% [IQR, 5.8–12.8] versus 2.1% [IQR, 1.5–2.2]; *P*=0.0028) (Figure 2B). Conversely, a trend towards an increase in lesional smooth muscle cell α-actin content was seen in ApoE^-/-^Irf5^-/-^ *mice* at 27 weeks of age (*P*=0.052; Figure IIA in the online-only Data Supplement).

### IRF5 Deletion in Hypercholesterolemic Mice Modulates Macrophage Markers in Atherosclerotic Lesions

Necrotic core development is strongly related to the behavior of myeloid cell populations in particular macrophages.^20^ IRF5 defines a proinflammatory macrophage phenotype.^8^ Both proinflammatory and alternatively activated macrophages have been identified in atherosclerotic lesions.^21,22^ Thus, we investigated whether IRF5 deletion affected the expression of macrophage markers within atherosclerotic plaques. Lesions were stained for the proinflammatory macrophage marker inducible nitric oxide synthase (iNOS), the alternatively activated macrophage marker CD206 (mannose receptor), and the lipid/heme driven Mox/Mhem macrophage marker heme oxygenase-1. Heme oxygenase-1 expression was significantly upregulated in the aortic root lesions of 15-week-old ApoE^-/-^Irf5^-/-^ compared with ApoE^-/-^ mice (*P*=0.002; Figure IIB in the online-only Data Supplement), as was lesional CD206 expression at 27 weeks of age (*P*=0.030; Figure 2C). No difference in lesional iNOS protein expression was seen at the time points studied by immunohistochemistry (Figure IIC in the online-only Data Supplement).

**Figure 3.**
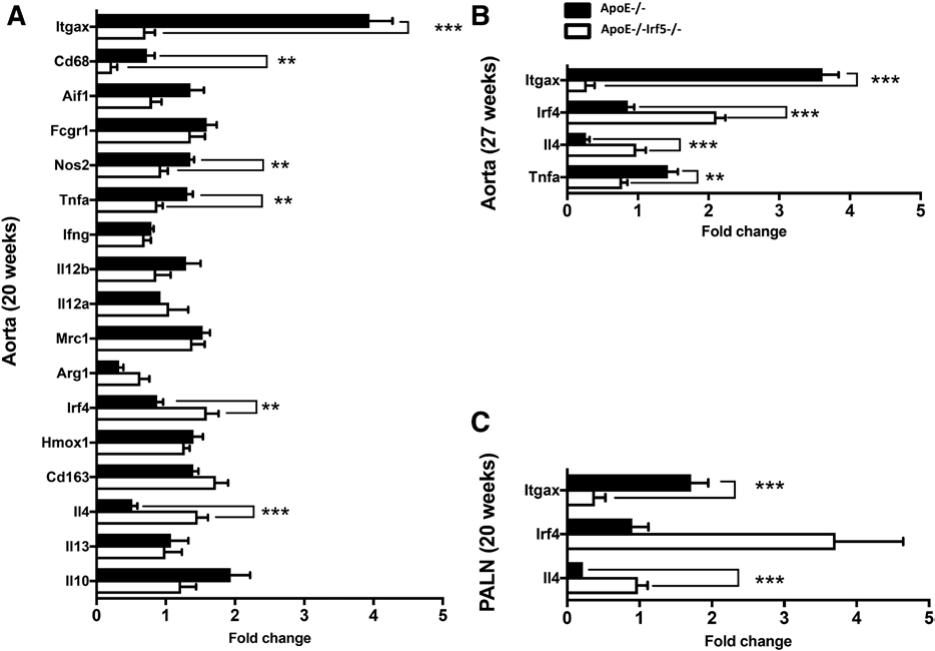
**Decreased gene expression of inflammatory and myeloid cell markers in ApoE^-/-^Irf5^-/-^ mice.** The aortic arches and PALNs of ApoE^-/-^ and ApoE^-/-^Irf5^-/-^ mice were collected and RNA extracted. Gene expression of inflammatory and myeloid cell markers was then assessed by reverse transcription polymerase chain reaction. **A** and **B**, Graphs show gene expression of selected genes in the aortic arch of ApoE^-/-^ (black bars) and ApoE^-/-^Irf5^-/-^ mice (white bars) at 20 (**A**) and 27 (**B**) weeks of age. **C**, Graph shows gene expression of selected genes in the PALN of ApoE^-/-^ (black bars) and ApoE^-/-^Irf5^-/-^ mice (white bars) at 20 weeks of age. Data are presented as fold change (n=8). Bars=mean+SEM. ApoE indicates apolipoprotein E-deficient; IRF, interferon regulatory factor; and PALN, para-aortic lymph nodes. **P<0.01; ***P<0.001.

To investigate the modification of myeloid cell programming in the lesions, gene expression levels of a bespoke panel of myeloid markers were quantified in the aortic arch of 20-week-old mice, the time point at which the maximum difference in lesion size between ApoE^-/-^ and ApoE^-/-^Irf5^-/-^ mice was observed. The levels of gene expression of *Itgax* (CD11c; *P*=1x10^−6^), *Cd68* (*P*=0.0016), *Nos2* (*P*=0.0016), and tumor necrosis factor*-α* (*P*=0.0017) were significantly reduced in the aorta of *ApoE*^-/-^*Irf5*^-/-^ compared with ApoE^-/-^ mice, whereas *Irf4* (*P*=0.0023) and *Il4* (*P*=5x10^−5^), were upregulated (Figure 3A). A similar pattern of gene expression was found in the aorta at 27 weeks of age (Figure 3B) and the PALNs (situated by the iliac bifurcation, a common site of atherosclerotic plaque development; Figure 3C).

### IRF5 Deficiency Reduces Shear Stress-Modulated Plaque Vulnerability

A perivascular shear stress-altering cast, which is surgically tied around the carotid artery of ApoE^-/-^ mice, has been shown to mimic human shear stress patterns and generates atherosclerotic lesions with TCFA morphology in mice. In this model, LSS induces macrophage-rich vulnerable plaques, and oscillatory shear stress (OSS) produces smooth muscle cell-rich stable plaques.^14^ We previously showed that IRF5 expression is enhanced at the LSS-exposed region of the carotid artery, in keeping with higher expression of inflammatory macrophage markers, including iNOS at this site.^15^

To investigate whether IRF5 modulates plaque size and composition in a murine model of TCFA, ApoE^-/-^, and ApoE^-/-^Irf5^-/-^ mice were fed a high-fat diet for 2 weeks before undergoing surgery to place the perivascular shear stress-modifying cast. Animals were euthanized and casts removed after 9 weeks. ApoE^-/-^Irf5^-/-^ mice fed a high-fat diet were found to be significantly heavier than *ApoE*^-/-^ mice (Table VII in the online-only Data Supplement) as previously published.^10^ However, no differences in serum cholesterol levels were detected between the 2 groups (Table VII in the online-only Data Supplement).

**Figure 4.**
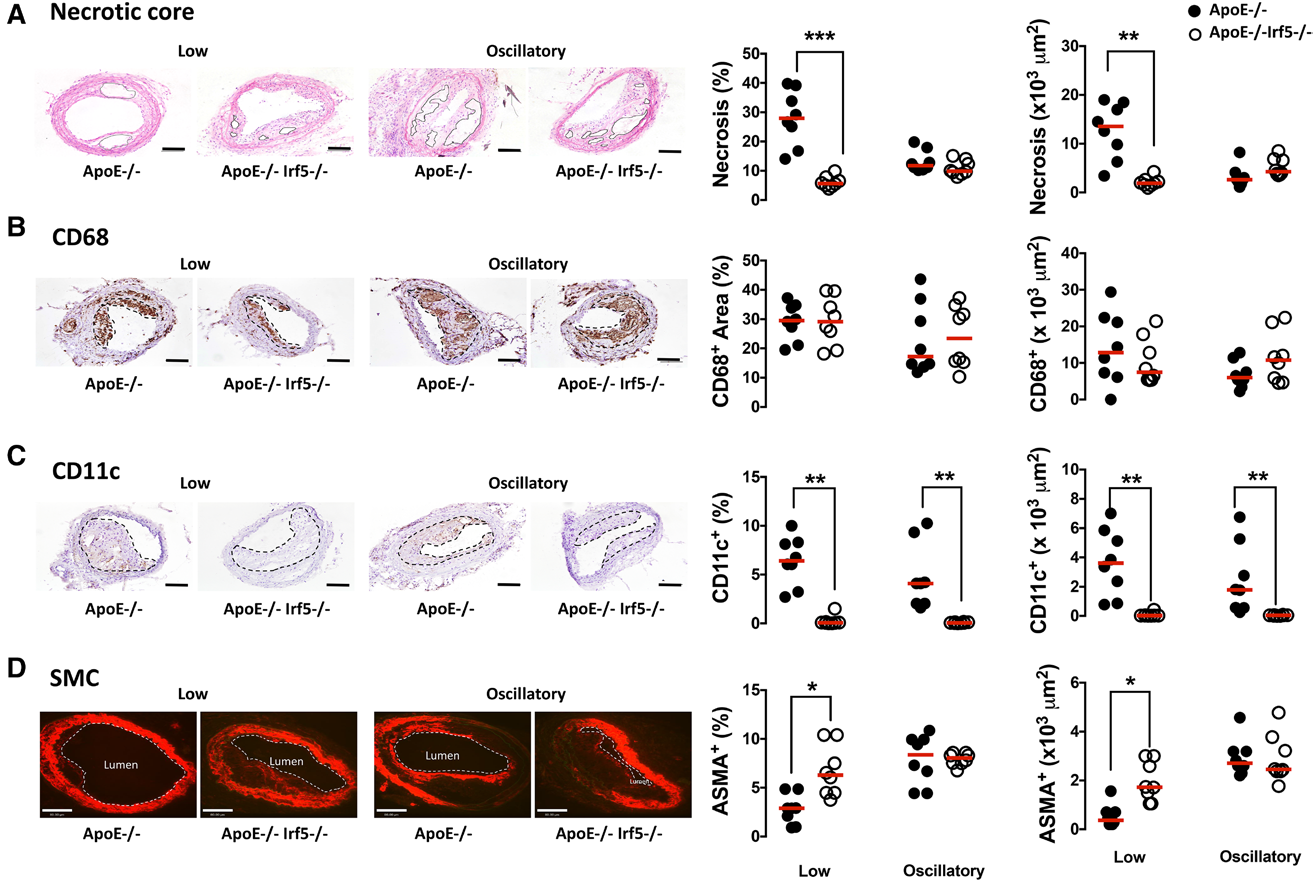
**IRF5 deficiency decreases necrotic core formation and CD11c expression in shear stress-modulated lesions.** ApoE^-/-^ (black circles) and ApoE^-/-^Irf5^-/-^ mice (white circles) were placed on a high-fat diet at 17 to 18 weeks of age. After 2 weeks, a perivascular shear stress-altering cast was surgically placed around the common carotid artery and left in place for 9 weeks. **A**, Representative photomicrographs of carotid artery sections stained with hematoxylin and eosin. Graphs show lesional necrotic core area (defined as anuclear, afibrotic, and eosin-negative areas) (x1000 μm^2^ and %). Solid lines show necrotic core area. **B**, Representative photomicrographs of carotid sections stained with an antibody against CD68 (brown staining) and hematoxylin. Graphs show lesion area staining positive (x1000 μm^2^ and %) for CD68. Dotted lines denote lesion area. **C**, Representative photomicrographs of carotid sections stained with an antibody against CD11c (brown staining) and hematoxylin. Graphs show lesion area staining positive (x1000 μm^2^ and %) for CD11c. Dotted lines denote lesion area. **D**, Representative photomicrographs of carotid sections with an antibody against smooth muscle cell α-actin (ASMA) (Cy3-red). Graphs show aortic root lesion area staining positive (x1000 μm^2^ and %) for ASMA. Dotted lines denote lumen. Bars=100μm in **A** through **C** and 80 μm in **D** (n=8). Each circle represents the mean positive area per individual mouse. Horizontal line denotes group median. ApoE indicates apolipoprotein E-deficient; IRF, interferon regulatory factor; and SMC, smooth muscle cell. *P<0.05; **P<0.01; ***P<0.001.

No statistically significant differences were shown in lesion size between the 2 genotypes after cast placement (Figure III in the online-only Data Supplement). The size of the necrotic core in the lesions induced by LSS was significantly smaller in ApoE^-/-^Irf5^-/-^ compared with ApoE^-/-^ mice (27.9% [IQR, 23–35.1] versus 5.65% [IQR, 4.93–6.83]; *P*=0.00062; Figure 4A), whereas no difference in necrotic core size of lesions in the OSS region was observed between the 2 genotypes. No differences were observed in the expression of CD68 (Figure 4B), iNOS, CD206, or heme oxygenase-1 (Table VIII in the online-only Data Supplement).

CD11c expression was almost abrogated in both LSS- and OSS-modulated regions of the ApoE^-/-^Irf5^-/-^ carotid compared with ApoE^-/-^ mice (LSS: 6.41% [IQR, 5.35–8.17] versus 0.07% [IQR, 0.045–0.097]; *P*=0.0036; OSS: 4.09% [IQR, 2.04–5.48] versus 0.06% [IQR, 0.027–0.125]; *P*=0.0037; Figure 4C). It is important to note that IRF5 deficiency increased the smooth muscle cell content in the LSS region (*P*=0.029; Figure 4D).

### IRF5 Deficiency Reduces CD11c Expression in the Aorta, Draining Lymph Nodes, and Bone Marrow Cultures

**Figure 5.**
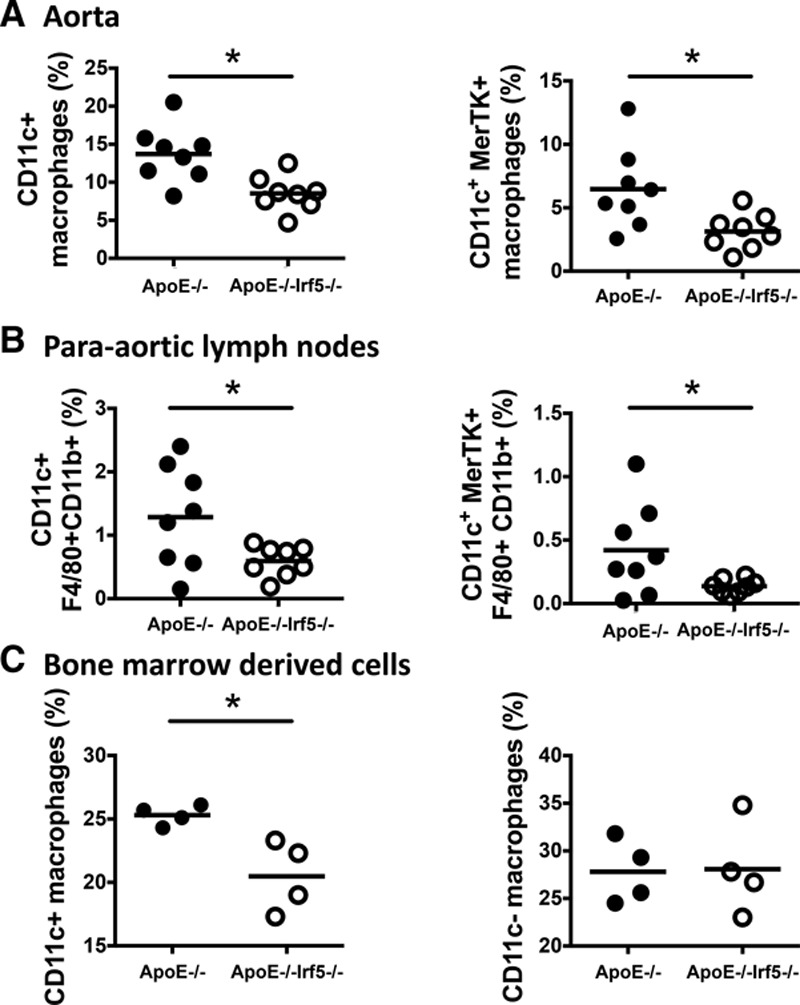
**IRF5 affects myeloid cell phenotype in the aorta, para-aortic lymph nodes, and in vitro bone marrow cultures.** Aortas, PALNs, and bones were harvested from 20- to 24-week-old ApoE^-/-^ and ApoE^-/-^Irf5^-/-^ mice. Single-cell suspensions were then stained with antibodies against myeloid cell markers and analyzed by flow cytometry. Dead cells and debris were excluded from the analysis, and cells were gated on CD45^+^ cells. **A**, Graphs show the numbers of aortic CD11c^+^ macrophages (gated as CD45^+^CD11b^+^F4/80^+^cells or as CD45^+^CD11b^+^F4/80^+^MerTK^+^), expressed as a percentage of CD45^+^ cells (n=8). **B**, Graphs show the numbers of CD11c^+^ macrophages in the PALNs, expressed as a percentage of CD45^+^ cells (n=8). **C**, Graphs show the numbers of CD11c^+^- and CD11c- expressing macrophages in GM-CSF-derived macrophage cultures in vitro, expressed as a percentage of CD45^+^ cells (n=4). Each circle represents an individual mouse. Horizontal line denotes group mean. ApoE indicates apolipoprotein E-deficient; IRF, interferon regulatory factor; MerTK, tyrosine-protein kinase Mer; and PALN, para-aortic lymph nodes. *P<0.05.

A reduction in CD11c expression observed by both immunohistochemistry and gene expression quantification emerged as the most striking change in myeloid markers induced by IRF5 deficiency in vivo. To assess whether differences in gene expression reflected changes in specific cell populations, 8-color flow cytometry was performed on aortas, PALNs, and in vitro GM-CSF-derived bone marrow-derived cell cultures from ApoE^-/-^ and ApoE^-/-^Irf5^-/-^ mice. In line with the gene expression data, *ApoE*^-/-^*Irf5*^-/-^ mice showed a significant decrease in CD11c-expressing F4/80^+^CD11b^+^ and F4/80^+^CD11b^+^MerTK^+^ macrophages in the aorta and PALNs (Figure 5A and 5B and Figures IV and V in the online-only Data Supplement) compared to ApoE^-/-^ mice. There was also a decrease in the comparatively small CD11b^+^ dendritic cell population (*P*<0.05; Figure VI in the online-only Data Supplement). However, there was no change in CD103^+^ dendritic cells in the aorta of ApoE^-/-^Irf5^-/-^ compared with ApoE^-/-^ mice (Figure VI in the online-only Data Supplement). Attenuation of CD11c^+^ macrophage frequencies in ApoE^-/-^Irf5^-/-^ compared with ApoE^-/-^ mice was also confirmed in GM-CSF-derived bone marrow cell cultures (*P*<0.05; Figure 5C).

**Figure 6.**
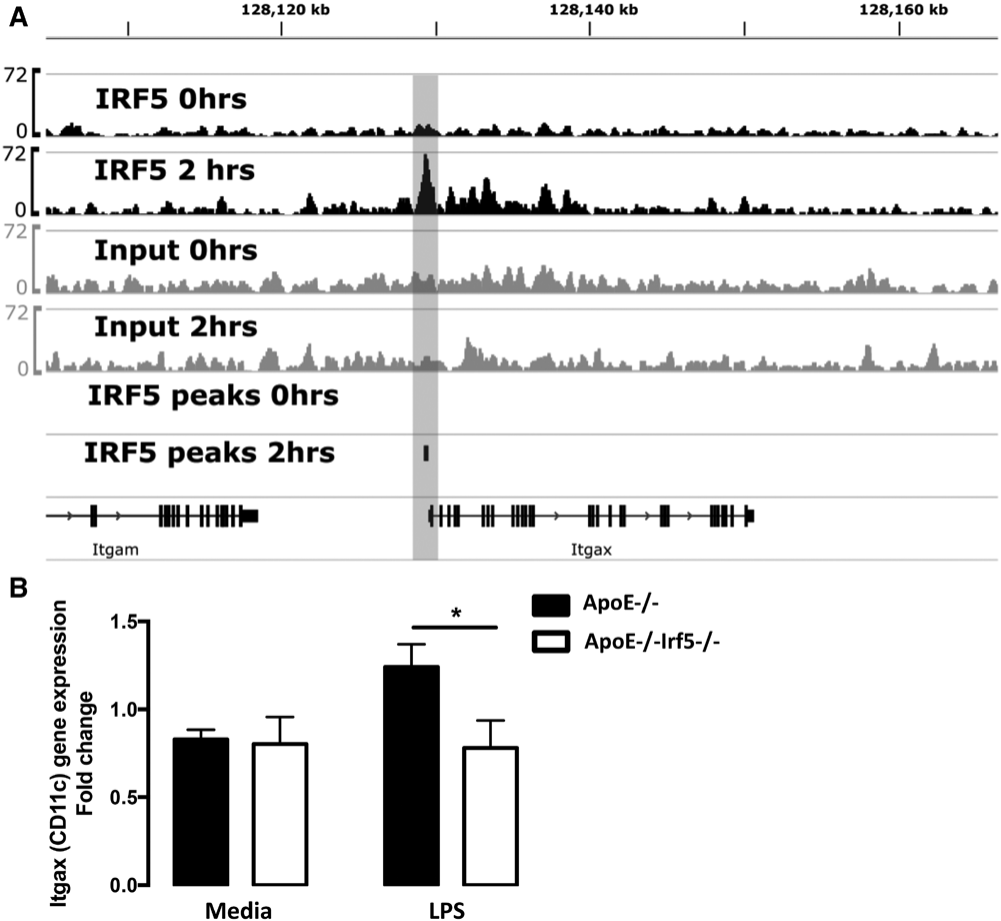
**IRF5 regulates CD11c expression.** **A**, Chromatin immunoprecipitation and next-generation sequencing (ChIP-seq) of IRF5 binding sites from unstimulated and LPS-stimulated GM-CSF bone marrow-derived cells revealed specific binding of the transcription factor to the promoter region of ITGAX in GM-CSF-cultured bone marrow-derived macrophages. Notably, the binding of IRF5 to the ITGAX gene loci is dependent on LPS stimulation, with recruitment of IRF5, as detected by peak calling with MACS2, occurring in stimulated macrophages but not in untreated cells. **B**, Graph shows the fold change of CD11c gene expression in unstimulated or LPS-stimulated GM-CSF bone marrow-derived cells from ApoE^-/-^ (black bars) or ApoE^-/-^Irf5^-/-^ (white bars) mice. CD11c gene expression is downregulated in GM-CSF-matured bone marrow-derived macrophages from IRF5-deficient mice upon LPS stimulation (n=6 to 10). Data are presented as mean±SEM. ApoE indicates apolipoprotein E-deficient; GM-CSF, granulocyte-macrophage colony-stimulating factor; IRF, interferon regulatory factor; Itgb3, integrin-β3; and LPS, lipopolysaccharide. *P<0.05.

Chromatin immunoprecipitation revealed specific binding of IRF5 to the promoter region of *Itgax* (Figure 6A). Notably, the binding of IRF5 to the CD11c gene loci was dependent on lipopolysaccharide (LPS) stimulation, with recruitment of IRF5, as detected by peak calling with MACS2, occurring in stimulated macrophages but not in untreated cells. This finding is in keeping with the key role of IRF5 as a stimulus-dependent transcription factor in the macrophage immune response. Next we aimed to investigate whether IRF5 regulates the expression of CD11c in GM-CSF-matured macrophages. The levels of *Itgax* (CD11c) expression were significantly reduced in LPS-treated GM-CSF-cultured bone marrow-derived macrophages deficient in IRF5 (*P*<0.05; Figure 6B).

Next we explored the coexpression of IRF5 and CD11c within human atherosclerotic plaques. The expression of IRF5 and CD11c was assessed histologically in human carotid plaques from the Carotid Plaque Imaging Project biobank. As shown in Figure VIIA in the online-only Data Supplement, IRF5 and CD11c were found to be coexpressed, and their coexpression appeared to be more marked in regions of the plaque that were adjacent to the necrotic core. Furthermore, the percentage of CD11c expression positively correlated with IRF5 expression (*r*=0.57, *P*<0.05; Figure VIIB in the online-only Data Supplement), suggesting that similar to murine atherosclerosis there may be a role for IRF5 in regulating CD11c expression in human atherosclerotic disease.

### IRF5 Deficiency Affects the Function of Myeloid Cell Subsets

Our data demonstrate that IRF5 deficiency in mice with hypercholesterolemia reduces both the intralesional necrotic area and CD11c^+^ myeloid cell content. Next, we explored the mechanistic relationship between these 2 observations by investigating whether IRF5 deficiency affected the functional behavior of myeloid cells in mice with hypercholesterolemia.

Bone marrow-derived cells were cultured in the presence of GM-CSF before being magnetically sorted into CD11c^+^ and CD11c^-^ populations. The cells were then left unstimulated or activated with LPS. We investigated whether *Irf5* deficiency modifies the phagocytosis and foam cell formation capacity of myeloid cells. The GM-CSF-cultured CD11c^+^ or CD11c^-^ bone marrow cells were incubated with fluorescently labeled acetylated human low-density lipoprotein or fluorescent microspheres to assess their phagocytosis efficiency. IRF5 deficiency did not affect the ability of the bone marrow-derived cells to take up fluorescently labeled acetylated human low-density lipoprotein and become foam cells, nor did it affect their ability to perform bead phagocytosis (Tables IX and X in the online-only Data Supplement).

**Figure 7.**
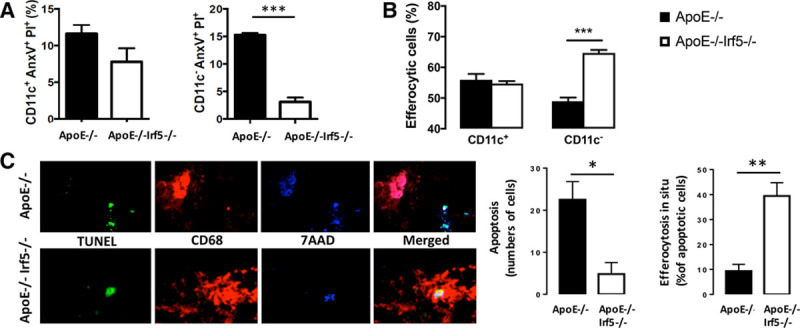
**IRF5 deficiency reduces cellular apoptosis and increases efferocytosis.** Bone marrow cells from ApoE^-/-^ (black bars) and ApoE^-/-^Irf5^-/-^ (white bars) mice 16 to 20 weeks of age were cultured in GM-CSF for 7 days, and their ability to undergo apoptosis and to perform efferocytosis was assessed. **A**, Graph shows percentage of CD11c^+^ and CD11c^-^ cells staining positive for propidium iodide and Annexin V after LPS stimulation and ultraviolet light exposure (n=8). **B**, Graph shows percentage of apoptotic Jurkat cell uptake by CD11c^+^ and CD11c^-^ macrophages (n=8). **C**, Representative images of perivascular flow-modifying cast-induced carotid lesions in ApoE^-/-^ and ApoE^-/-^Irf5^-/-^ mice stained with an antibody against CD68 (red) and TUNEL stained (green) and DNA (blue). Graphs show number of apoptotic cells (left) and percentage (right) of apoptotic cells undergoing efferocytosis in situ (n=4 to 5). Data are presented as mean±SEM. Black bars, *ApoE*^-/-.^. White bars, *ApoE*^-/-^*Irf5*^-/-^. ApoE indicates apolipoprotein E-deficient; and IRF, interferon regulatory factor. *P<0.05; **P<0.01; ***P<0.001.

The amount of cell apoptosis is an important factor in necrotic core formation.^23^ Apoptosis was induced with ultraviolet light, cells were stained with propidium iodide and Annexin V, and apoptosis levels were measured by flow cytometry. It is interesting to note that late apoptosis was decreased in ApoE^-/-^Irf5^-/-^ CD11c^-^ cells after activation with LPS (*P*<0.0001; Figure 7A and Table XI in the online-only Data Supplement).

Finally, the ability to perform apoptotic cell removal (efferocytosis) was examined by incubating the bone marrow-derived cells with fluorescently labeled apoptotic Jurkat cells as previously published.^18^ The efficiency of efferocytosis by GM-CSF differentiated ApoE^-/-^Irf5^-/-^ CD11c^-^ cells was ≈30% higher than that of differentiated ApoE^-/-^ CD11c^-^ cells (*P*<0.0001; Figure 7B).

To investigate whether the effect of IRF5 deficiency on efferocytosis was also observable in situ, carotid plaque sections were costained for apoptosis with TUNEL stain and CD68 using previously described methods.^24^ In support of the in vitro data, the frequency of apoptotic cells was decreased in situ in ApoE^-/-^Irf5^-/-^ mice (*P*=0.04; Figure 7C). Conversely, the percentage of apoptotic cells undergoing efferocytosis was enhanced in plaque tissue obtained from IRF5-deficient ApoE^-/-^ mice (*P*=0.001; Figure 7C).

In summary, IRF5 deficiency promotes the viability of CD11c^-^ GM-CSF-derived bone marrow macrophages and their ability to perform efferocytosis, but it does not affect their ability to phagocytose lipid or beads.

### IRF5 Suppresses Efferocytosis-Regulating Genes In Vitro and In Situ

To further explore how IRF5 may interfere with myeloid cell capacity to perform efferocytosis, gene expression of the most characterized receptors for efferocytosis, the TAM (Tyro3, Axl, and Mer) receptors tyrosine-protein kinase Mer (MerTK), Axl, and Tyro3, as well as Itgb3 (which forms the α_v_β_3_ receptor with alpha 5 integrin), were analyzed in GM-CSF-matured bone marrow-derived myeloid cell cultures.

**Figure 8.**
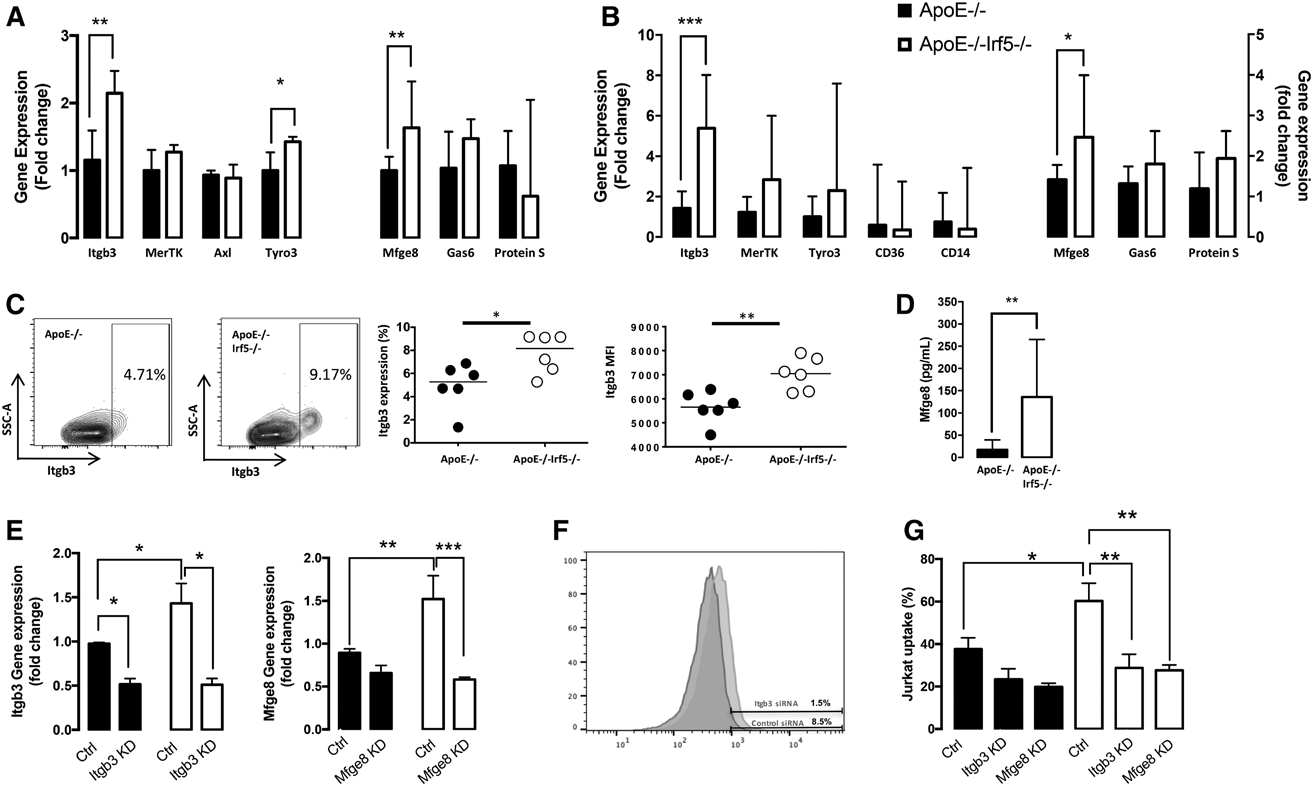
**IRF5 deficiency increases efferocytosis by CD11c^-^ cells by an upregulation of Itgb3 and Mfge8.** **A**, Gene expression (shown as fold change) of efferocytosis regulating receptors (*Itgb3*, *MerTK*, *Axl*, and *Tyro3*) and efferocytosis-regulating bridging molecules (Mfge8, Gas6, and Protein S) in GM-CSF-matured macrophage cell cultures from ApoE^-/-^ (black bars) and ApoE^-/-^Irf5^-/-^ (white bars) mice 16 to 20 weeks of age (n=6 to 8). Data presented as median and IQR. **B**, Gene expression (shown as fold change) of efferocytosis-regulating receptors (*Itgb3*, *MerTK*, *Tyro3*, *CD36*, and *CD14*) and receptor ligands (*Mfge8*, *Gas6*, and *Protein S*) in the aorta of 20-week-old ApoE^-/-^ and ApoE^-/-^Irf5^-/-^ mice (n=5 to 8). Data presented as median and IQR. **C**, CD11c^-^ GM-CSF-matured macrophages from *ApoE*^-/-^ and *ApoE*^-/-^*Irf5*^-/-^ mice were stained with an antibody against Itgb3. Representative plots show Itgb3 expression on CD11c^-^ cells and graphs show numbers (left) of CD11c− cells expressing Itgb3 and the median fluorescent intensity (MFI) of Itgb3 staining (right) (n=6). Horizontal bars denote group median. **D**, Bone marrow-derived cells from ApoE^-/-^ and ApoE^-/-^Irf5^-/-^ mice were cultured in GM-CSF for 7 days, and Mfge8 released into the supernatant was assessed by ELISA (n=8). Data are presented as median and IQR. **E**, Gene expression level (fold change) of Itgb3 (left) and Mfge8 (right) in GM-CSF-matured macrophages from ApoE^-/-^ (black bars) and ApoE^-/-^Irf5^-/-^ (white bars) mice after transfection with siRNA against Itgb3, Mfge8, or a control siRNA. Graphs show mean and SEM (n=3). **F**, Histogram overlay of Itgb3 protein expression, as assessed by flow cytometry, on GM-CSF-matured macrophages after transfection with siRNA against Itgb3 (red) or control (blue). **G**, Percentage of apoptotic Jurkat cell uptake by GM-CSF-cultured macrophages from ApoE^-/-^ (black bars) and ApoE^-/-^Irf5^-/-^ (white bars) mice after transfection with siRNA against Itgb3, Mfge8, or a control siRNA. Graphs show mean and SEM (n=4). ApoE indicates apolipoprotein E-deficient; Ctrl, control; GM-CSF, granulocyte-macrophage colony-stimulating factor; IRF, interferon regulatory factor; Itgb3, integrin-β3; MerTK, tyrosine-protein kinase Mer; and Mfge8, milk fat globule-epidermal growth factor 8 protein. *P<0.05; **P<0.01; ***P<0.001.

GM-CSF-matured cells from ApoE^-/-^Irf5^-/-^ mice displayed significantly higher gene expression of Itgb3 compared with cells from ApoE^-/-^ mice (*P*=0.0037; Figure 8A). A small increase in Tyro3 expression was also found (*P*=0.013; Figure 8A), whereas no effect on the gene expression of MerTK or Axl was observed. Since in vitro-matured cells may not always reflect the phenotype of cells matured in vivo, we next assessed the expression of efferocytosis-regulating genes in the aortas of ApoE^-/-^ and ApoE^-/-^Irf5^-/-^ mice. Again, Itgb3 expression was significantly higher in the aortas of ApoE^-/-^Irf5^-/-^ compared with ApoE^-/-^ mice (*P*=0.0006; Figure 8B). The increase in Itgb3 gene expression in IRF5-deficient ApoE^-/-^ mice was also confirmed at the level of cell surface protein expression using flow cytometry (*P*<0.05; Figure 8C). It is important to note that the increase in Itgb3 was restricted to CD11c^-^ macrophages, in keeping with the results of the functional efferocytosis assays.

We next assessed gene expression of the ligand of α_v_β_3_, Mfge8, and the ligands for the TAM receptors: growth arrest-specific 6 and protein S in GM-CSF-derived bone marrow myeloid cells. The gene expression of Mfge8 was found to be significantly higher in ApoE^-/-^Irf5^-/-^ compared with ApoE^-/-^ mice (*P*=0.0085; Figure 8A). This increase in Mfge8 gene expression was also present in the aorta of ApoE^-/-^Irf5^-/-^ compared with ApoE^-/-^ mice (*P*=0.01; Figure 8B). No differences in gene expression of the TAM receptors ligands growth arrest-specific 6 or protein S were found between *ApoE*^-/-^ and *ApoE*^-/-^*Irf5*^-/-^ mice in vitro or in situ (Figure 8A and 8B).

Since Mfge8 acts as a ligand between α_v_β_3_ and phosphatidylserine on apoptotic cells, the secretion of the protein is crucial for its effects in the lesion. Herein, Mfge8 levels were analyzed in supernatants of the in vitro bone marrow cell cultures. Mfge8 levels were found to be significantly higher in the supernatants from *ApoE*^-/-^*Irf5*^-/-^ compared with *ApoE*^-/-^-derived cells cultured in GM-CSF (*P*=0.005; Figure 8D).

To investigate whether the enhanced capacity to perform efferocytosis in Irf5-deficient macrophages was Itgb3 or Mfge8 dependent, additional knockdown experiments were performed. Using siRNA, Mfge8 and Itgb3 were knocked down in GM-CSF-derived bone marrow-derived macrophages from Irf5-deficient and Irf5-competent ApoE^-/-^ mice. The increase in Itgb3 and Mfge8 gene expression in Irf5-deficient compared with Irf5-competent ApoE^-/-^ mice was confirmed in the control siRNA conditions (*P*<0.05 and *P*<0.01, respectively; Figure 8E). Next, the effect of siRNA knockdown was assessed using quantitative reverse transcription polymerase chain reaction. The gene expression of both Itgb3 and Mfge8 were confirmed to be significantly suppressed in IRF5-deficient macrophages after siRNA transfection (*P*<0.05 and *P*<0.001, respectively; Figure 8E). Using flow cytometry, knockdown of Itgb3 was confirmed in IRF5-deficient macrophages at the protein level (Figure 8F).

To assess whether the knockdown of Itgb3 or Mfge8 affected the efferocytosis capacity of Irf5-deficient macrophages, the in vitro efferocytosis assay was repeated. A significantly increased efferocytosis capacity in the uptake of apoptotic Jurkat cells in the Irf5-deficient group compared with the Irf5-competent group was confirmed in the siRNA control groups (37.7% ± 5.4% versus 60.3% ± 8.3%, *P*<0.05; Figure 8G). Knockdown of either Itgb3 or Mfge8 decreased the efferocytic ability and thus reversed the in vitro phenotype of the IRF5-deficient macrophages to the level of those from IRF5-competent mice (60.3% ± 8.3% versus 28.8% ± 6.4%, *P*<0.01 and 60.3% ± 8.3% versus 27.6% ± 2.6%, *P*<0.01; Figure 8G).

## Discussion

Myeloid cells are powerful contributors to the development and complications of atherosclerosis. IRF5 is a master regulator of myeloid cell function and programming in mouse and human.^8^ We show that IRF5 deletion in ApoE^-/-^ mice reduces aortic atherosclerotic lesion size and also decreases the expression of the integrin CD11c in both macrophages and atherosclerotic lesions. In sharp contrast, IRF5 deficiency enhances both the expression of the integrin Itgb3 and its bridging ligand Mfge8 and the ability of myeloid cells to perform efferocytosis. This finding leads to a significant decrease in the necrotic core size in aortic atherosclerosis and LSS-induced TCFA lesions, demonstrating that IRF5 is instrumental in necrotic core formation.

### IRF5 Affects the Lesional Content of CD11c^+^ Macrophages

CD11c^+^ myeloid cells are present in the arterial wall at athero-susceptible sites exposed to alterations in hemodynamic stress, such as curvatures and bifurcations.^25^ CD11c expression by monocytes is upregulated by hypercholesterolemia,^26^ and depletion of CD11c^+^ cells has been shown to reduce atherogenesis in ApoE^-/-^ mice.^19,26^ Collectively, these studies indicate the existence of proatherogenic CD11c^+^ myeloid cell populations.

In the present study, we observed a significant reduction in lesional CD11c staining in the absence of IRF5 in both hypercholesterolemia-induced and LSS-induced murine atherosclerosis models. Using flow cytometry to pinpoint the specific cell subsets affected by IRF5 deficiency, we were able to show a reduction in CD11c^+^ macrophages in the aortic lesions and draining lymph nodes. The reduction in CD11c expression was confirmed at the gene level in aortas and PALNs from IRF5-deficient ApoE^-/-^ mice. Loss of CD11c was also evident in vitro in IRF5-deficient GM-CSF-matured macrophages. In a chromatin immunoprecipitation sequencing survey of genome-wide IRF5 binding sites in GM-CSF cultured bone marrow-derived macrophages, a specific binding site was revealed in the promoter region of the *Itgax* (CD11c) gene. In addition, the level of CD11c gene expression was significantly reduced in macrophages derived from IRF5-deficient ApoE^-/-^ mice upon LPS stimulation. These data suggest that on activation IRF5 directly regulates the gene expression of CD11c in macrophages.

We have previously shown that IRF5 promotes an inflammatory macrophage phenotype by direct regulation of selected cytokines and surface receptors,^8,10,11,17^ and it is now widely used as a marker of inflammatory macrophages.^27,28^ This study adds Itgax (CD11c) to the list of IRF5-regulated markers that define an inflammatory macrophage phenotype. The involvement of the integrin CD11c in cell adhesion,^29^ monocyte/macrophage recruitment,^26^ and modulation of IL-1 production^30^ is well documented in the context of atherosclerosis.

The expression of CD11c has previously been shown to associate with proinflammatory macrophage phenotype in obesity.^31^ Loss of aortic CD11c gene expression in IRF5-deficient mice was associated with a reduction in the aortic expression of proinflammatory genes such as iNOS and tumor necrosis factor-α, and an increase in genes associated with alternatively activated macrophages such as interleukin 4and IRF4, indicating that IRF5 is responsible for a shift toward a proinflammatory myeloid cell programming in atherosclerosis. IRF5 induced other changes in macrophage phenotype in a time-dependent manner. At early stages of disease, heme oxygenase-1 expression was increased in lesions of ApoE^-/-^Irf5^-/-^ compared with ApoE^-/-^ mice, whereas at more advanced stages, CD206 expression was increased in ApoE^-/-^Irf5^-/-^ lesions. The change in CD206 expression fits with the observed increase in gene expression of M2-inducing interleukin 4 and IRF4 in the aorta. Our data indicate that CD11c expression is the hallmark of an IRF5-dependent subset of macrophages with a detrimental role in atherosclerosis.

GM-CSF is a strong inducer of IRF5 expression.^8^ Our data show that IRF5 deletion decreases the representation of CD11c^+^ bone marrow-derived myeloid cells after differentiation with GM-CSF. This observation is in keeping with previous studies reporting that GM-CSF has an important role in the maintenance of CD11c^+^ myeloid cells in atherosclerosis.^32^

In support of our findings in murine atherosclerosis, a significant association between IRF5 and CD11c expression was also found in human atherosclerotic tissue (Figure VIIA in the online-only Data Supplement). IRF5 gene expression has previously been shown to be increased in human atherosclerotic tissue compared with control tissue, but less is known about the potential effects of IRF5 activity in human plaque biology.^33^ The present findings support a role for IRF5 as an important transcription factor, potentially supporting an inflammatory macrophage subset identifiable by the expression of CD11c in human disease.

Collectively, our data indicate that IRF5 is an important factor for the maintenance of CD11c^+^ myeloid cell populations within atherosclerotic lesions and also directly regulates the expression of CD11c on macrophages.

### IRF5 Increases Necrotic Core Size by Impairing Myeloid Cell Efferocytosis

The association between IRF5 and efferocytosis has not been previously described. In the present study, we show that IRF5 deficiency is associated with decreased necrotic core size, in both a standard hypercholesterolemia-driven model and a shear stress-modulating surgical model known to form TCFA.^15^ The necrotic core in atherosclerotic plaques arises because of a combination of macrophage apoptosis and defective efferocytosis (uptake of apoptotic cells).^5^ The capacity of macrophages to perform efferocytosis and phagocytosis is thought to be broadly dependent on their activation and polarization status, with efferocytosis being inhibited by tumor necrosis factor-α^34,35^ and enhanced by interleukin 4/interleukin 13 and peroxisome proliferator-activated receptor-γ signaling.^21,36^ The dysregulated inflammation present in advanced lesions is thought to play a role in disabling efferocytosis.^20^ However, the specific transcriptional regulators impairing efferocytosis in atherosclerosis are poorly defined.

IRF5 deletion uncovered a complex interaction between CD11c expression and myeloid cell capacity to perform efferocytosis. Isolated CD11c^-^ cells from GM-CSF-derived bone marrow cultures from IRF5-deficient ApoE^-/-^ mice performed efferocytosis to a significantly greater extent than CD11c^-^ cells from IRF5-competent ApoE^-/-^ mice. Furthermore, IRF5 deletion selectively affected macrophage efferocytosis and did not interfere with phagocytosis or lipid uptake functions, suggesting that IRF5 plays a selective role in efferocytosis. Our data support the concept that macrophage programming consists of modules that can be activated independently of others rather than an en bloc phenotype.

The mechanism of efferocytosis is dependent on specific receptors and bridging molecules and is distinct from phagocytosis. Efferocytosis prevents secondary necrosis and is crucial for regulating and resolving the inflammatory response in atherosclerotic lesions.^20^ Because IRF5 clearly affected the ability of CD11c^-^ cells to perform efferocytosis, we sought to identify potential efferocytosis pathways that Irf5 may regulate. MerTK is a member of the TAM receptor tyrosine kinases that have important functions in efferocytosis and regulation of innate immunity.^37^ Defective efferocytosis induced by the deletion of MerTK accelerates atherosclerosis and necrotic core formation.^24^ However, in our study, MerTK^+^ macrophages were decreased in lesions in ApoE^-/-^Irf5^-/-^ mice. This finding is in keeping with a recent article by Cai et al,^7^ which highlighted that proteolytic cleavage of MerTK occurs in atherosclerosis and supports necrotic core formation. The lack of enhancement of MerTK protein expression in IRF5-deficient mice suggested that other efferocytosis pathways were at play.

The α_v_β_3_ integrin is known to be a powerful mediator of efferocytosis through binding to phosphatidylserine on apoptotic cells by the bridging molecule Mfge8. Itgb3 is one of the regulating parts of the α_v_β_3_ integrin.^38^ Mfge8 is a glycoprotein secreted by macrophages that binds strongly to cells expressing α_v_β_3_ integrin. Depleting Mfge8 inhibits efferocytosis, and Mfge8 deficiency in atherosclerosis models accelerates atherosclerosis and leads to the accumulation of apoptotic cells.^38,39^ However, the role of Itgb3 on atherosclerotic efferocytosis has been less well described, and a role for IRF5 in regulating Itgb3 has not previously been shown. Both Itgb3 and its ligand Mfge8 were significantly upregulated in both the aorta and GM-CSF-derived cells from IRF5-deficient ApoE^-/-^ mice. Moreover, when Itgb3 and Mfeg8 were knocked down, the efferocytic phenotype of the IRF5-deficient cells was reversed, suggesting that the Mfeg8−Itgb3 pathway was responsible for the increased ability of IRF5-deficient macrophages to perform efferocytosis. In summary, we provide evidence for an important role of *Irf5* in impairing efferocytosis through the reduction of expression of Itgb3 and Mfge8.

Deletion of IRF5 might affect intralesional macrophage content through mechanisms other than efferocytosis. Macrophages from IRF5-deficient ApoE^-/-^ mice were resistant to apoptosis in vitro, and this observation is supported by intralesional TUNEL staining. In keeping with this observation, we observed a small increase in the CD68 immunopositive area percentage across all time points. Thus, the effect of IRF5 deletion on lesional CD68^+^ cell content could possibly be explained by the complex role of IRF5 in cell survival and efferocytosis.

Our data reveal that IRF5 deletion has a significant effect on macrophage phenotype and function in atherosclerosis. IRF5 is expressed by monocytes, macrophages, conventional dendritic cells, plasmacytoid dendritic cells, B cells, and some stromal cells.^40^ Although the expression of IRF5 within mouse and human atherosclerotic lesions appeared to be confined to myeloid cells, our data (obtained with a global genetic deletion of IRF5) cannot exclude indirect effects arising from the deletion of IRF5 in other cell types and thus is a limitation of our study.

## Conclusions

In summary, the transcription factor *Irf5* has a detrimental effect in atherosclerosis by affecting plaque formation and stability through the modulation of macrophage phenotype and function. IRF5 activation directly regulates expression of the integrin CD11c on macrophages and promotes the maintenance of proinflammatory CD11c^+^ macrophages within the lesions. Moreover, IRF5 actively contributes to the impairment of efferocytosis, and its deletion ameliorates the formation of the necrotic core by the upregulation of the integrin Itgb3 and its ligand Mfge8 (Figure VIII in the online-only Data Supplement). Our study highlights the existence of equilibrium between CD11c^+^ and CD11c^-^ Itgb3^+^ macrophage subsets within atherosclerotic lesions, which significantly impacts the formation of the necrotic core.

## Sources of Funding

The research leading to these results has received funding from the British Heart Foundation Center of Research Excellence, Imperial College London, the European Commission under the Seventh Framework Program (FP7/2007–2013; contract no. 201668; AtheroRemo and HEALTH.2012-1.2-1; contract no. 305739 RiskyCAD), The Kennedy Trustees, The Swedish Heart and Lung foundation (20150277), The Swedish Research Council (2015-00582), the Swedish Society of Medicine (SLS-500141), Skåne University Hospital funds, Region Skåne Research funds, and the Novo Nordisk Foundation (grant no. NNF15CC0018346).

## Disclosures

None.

## Supplementary Material
