# Supplementary material for: Interferon Regulatory Factor 5 Controls Necrotic Core Formation in Atherosclerotic Lesions by Impairing Efferocytosis
Source: Circulation. 2017 Sep 19;136(12):1140–54. doi: 10.1161/CIRCULATIONAHA.117.027844 (PMC5598917; doi:10.1161/CIRCULATIONAHA.117.027844)
Supplement: Supplementary file 1 [file cir-136-1140-s001.pdf]

SUPPLEMENTAL MATERIAL

Expanded Materials and Methods

Atherosclerosis time-course

Mice were weaned at 4 weeks of age and fed a standard chow diet for the duration of the experiment. At 15, 20 or 27 weeks of age, $ApoE^{-/-}$ and $ApoE^{-/-}Irf5^{-/-}$ mice were euthanized with a barbiturate overdose and blood collected by cardiac puncture. Hearts were perfused *in situ* with saline via a cannula inserted into the left ventricle (outflow via an incision in the right atrium) and then the top halves of the heart were frozen in OCT embedding medium. The aorta and para-aortic lymph nodes were snap frozen for RNA extraction. Terminal blood was centrifuged at 6000rpm for 30 minutes at 4°C and serum collected. All tissues were stored at -80°C.

Surgical placement of a perivascular flow-modifying cast

Mice were placed on a cholate-free high fat diet (diet W) from Special Diets Services (Essex, UK) at 17-19 weeks of age. Two weeks later, mice were anaesthetised and a perivascular cast was tied around the right common carotid artery as previously described[1]. Nine weeks after cast placement, mice were euthanized and lesion development assessed as described.

Aortic root atherosclerotic lesion measurement

Five micrometer cryosections were taken of the aortic root for the entire region of the valve leaflets and every 20th section (100µm) was stained with Oil Red O and counterstained with hematoxylin. Aortic root sections were coded and analyzed blind. Images were captured under identical microscope, camera and light conditions using an Olympus BX51 osteometric brightfield & fluorescence microscope (Olympus). Quantification was performed by drawing around the atherosclerotic lesions and the aortic wall using Clemex Vision Lite version 5.0 (Clemex, Longueuil, Canada). Absolute values for cross-sectional area were obtained by calibrating the software using an image of a micrometer slide taken at the same magnification. The individual lesion areas per aortic root section were averaged to obtain the mean lesion area per mouse. The lesion area fraction was calculated by dividing the mean lesion area by the mean area of the aortic wall and expressed as a percentage.

Measurement of lesion formation in perivascular cast-induced injury

Nine weeks following cast placement, mice were euthanized, terminal blood collected via cardiac puncture and the vasculature perfused with 0.9% w/v saline. The injured carotid was dissected out and frozen at -80°C in Optimal cutting temperature (OCT) compound (ThermoScientific, Runcorn, UK). Serial 5µm cryosections were taken through the entire length of the carotid artery. Staining of the elastic lamina was performed using the Accustain kit (Sigma-Aldrich Inc., St. Louis, USA) according to the manufacturer's instructions. Measurement of lesion and vessel areas was performed using ProgRes CapturePro image analysis software (version 2.5.2.0, Jenoptik, Germany). The area between the internal and external elastic lamina was taken as the medial area and the intimal area was calculated by subtracting the lumen area from the internal elastic lamina area. The intimal medial ratio (IMR) was then calculated by dividing the intimal area by the medial area.

Hematoxylin and Eosin (H+E) staining and necrosis quantification

H+E staining was performed using a Tissue Tek Prisma/Film automated slide stainer (Sakura, Japan). Slides were fixed in 10% buffered formalin for 10 minutes, rinsed in distilled water and then stained with Harris hematoxylin for 8 minutes, washed in running tap water for 5 minutes, and differentiated with 0.3% acid alcohol for 2 minutes and rinsed in distilled water. The slides were counterstained with eosin for 2 minutes before being rinsed in distilled water. Necrotic areas (defined as being H+E free) were quantified using Clemex Vision Lite version 5.0. Only areas larger than 3000μm^2 in the aortic root and 300μm^2 in the carotid were included in the analysis. Absolute values were obtained by calibrating the software using an image of a micrometer slide taken at the same magnification. Necrotic lesion area was calculated by dividing the necrosis area by the lesion area and expressing it as a percentage.

Murine Immunohistochemistry

Immunohistochemistry was performed on 5µm cryosections using standard avidin biotinylated enzyme complex (ABC) methods as previously published[2]. In brief, sections were fixed in ice-cold acetone before incubation with 10% normal rabbit or goat serum for one hour. Following a wash in PBS, endogenous avidin and biotin were blocked using Vector avidin/biotin blocking kit (Vector labs, Peterborough, UK) according to manufacturer's instructions. Sections were then incubated with primary

antibodies against CD11c (BD Biosciences 2.5μg/mL), CD68 (AbD Serotec, 5μg/ml), iNOS (Abcam, 1μg/mL), CD206 (AbD Serotec, 5μg/mL), IRF5 (Abcam, 0.5μg/mL) or HO-1 (Abcam, 5μg/mL) for 45 minutes at room temperature, followed by relevant biotinylated secondary antibodies. Following blocking of endogenous peroxidase activity with 0.3% hydrogen peroxide, sections were incubated with avidin and biotinylated horseradish peroxidase macromolecular complexes using Vectastain Elite ABC kit (Vector Labs) according to manufacturer's instructions. Bound peroxidise was detected using 3,3'- diaminobenzidine (DAB) and nuclei counterstained with hematoxylin. Staining using an appropriate isotype-matched control was performed on a consecutive section as a control. Smooth muscle cell staining was performed using an antibody against alpha smooth muscle actin conjugated to Cy3 (Sigma 5μg/mL) and DAPI counterstaining of nuclei.

Immunofluorescence staining

Dual staining of aortic root sections with antibodies against IRF5 and CD68, CD11c or α-smooth muscle actin was performed using tyramide signal amplification (TSA) using a biotin-TSA kit as per manufacturer's instructions (Perkin Elmer). In brief, 5μm cryosections were fixed in ice-cold acetone for 5 minutes before endogenous peroxidase activity was blocked with 0.3% hydrogen peroxide in PBS for 15 minutes. Endogenous avidin and biotin activity were then blocked using the Vector avidin/biotin blocking kit. Following blocking with 20% normal goat serum for one hour, sections were blocked with TNB blocking buffer (0.1M Tris-HCl pH 7.5, 0.15M NaCl, 0.5% blocking buffer as supplied in kit) for 30 minutes before incubation with an antibody against IRF5 (Abcam, 0.5μg/mL) for 45 minutes. Following washing, sections were incubated with relevant biotinylated secondary antibodies then incubated with streptavidin-HRP for 30 minutes. Biotinyl tyramide working solution was then added for 5 minutes followed by streptavidin-Alexa Fluor 488 (Life Technologies). Sections were then re-blocked and stained with an antibody against CD68 (AbD Serotec, 5μg/mL) or CD11c (AbD Serotec 20μg/mL) and an Alexa Fluor 568 conjugated secondary antibody and DAPI (Life technologies). Alternatively, slides were stained with an antibody against alpha smooth muscle actin conjugated to Cy3 (Sigma 5μg/mL) and DAPI. Slides were viewed on an Ultraview confocal microscope (PerkinElmer Life Sciences, Cambridge, UK).

Quantification of murine immunohistochemical staining

For all quantification, images were captured under identical microscope, camera and light conditions, coded and analyzed blind. Aortic root lesion area staining positive for a given marker was quantified using Clemex Vision Lite version 5.0. Using the image analysis software, positive staining was detected and lesion area measured. Absolute values were obtained by calibrating the software using an image of a micrometer slide taken at the same magnification. Lesion area fraction staining positive was calculated by dividing the area staining positive by the lesion area and expressing it as a percentage.

Analysis of serum cholesterol

Total serum cholesterol levels were measured enzymatically using Infinity Total Cholesterol (Thermo Scientific), according to the manufacturer's instructions. A calibration serum (Randox Laboratories) with a known cholesterol concentration was used as a reference.

In vitro bone marrow cell culture

For *in vitro* experiments, bone marrow cells were isolated from tibia and femurs of 16-21 week old ApoE$^{-/-}$ and ApoE$^{-/-}$Irf5$^{-/-}$ mice. The cells were cultured for 7 days in Roswell Park Memorial Institute (RPMI) 1640 medium containing 25 mM hepes (Lonza) supplemented with 10% fetal bovine serum (FBS; Labtech), 2.5µg/mL Ampotericin-B, 100U/mL penicillin, 100µg/mL streptomycin and 50µM 2-mercaptoethanol (all Gibco). The cells were cultured in the presence of 20ng/mL GM-CSF (Peptrotech). In some experiments, GMCSF-derived bone marrow cells underwent magnetic cell separation with CD11c MicroBeads (Miltenyi Biotec) and CD11c+ and CD11c- bone marrow derived cells were cultured separately. Cells were either left unstimulated or stimulated with 100 ng/mL E. Coli derived LPS (Enzo Life Sciences, serotype: EH100 Ra) for 24 hours.

In situ efferocytosis assay

Sections of cast-induced carotid lesions were stained for apoptosis and DNA using the BrdU-Red DNA Fragmentation kit (TUNEL, ab66110 Abcam, Cambridge, UK) according to manufacturer's instructions. Slides were counterstained for macrophages using an antibody against CD68 (Alexa Fluor 647, AbD Serotec). The most stenotic part of each section was imaged using an Olympus FV1200 IX83

confocal system. Images were analyzed using ImageJ (1.50I, NIH, USA) and Photoshop CS6 (13.0.1x32, Adobe Systems Inc, USA). TUNEL+ apoptotic cells surrounded by a CD68+ cell were counted as cells undergoing efferocytosis, as previously described[3]. TUNEL+ apoptotic cells not surrounded by a CD68+ cell were counted as apoptotic cells not undergoing efferocytosis. All analysis was performed by 2 independent assessors blinded to the experiment details.

Apoptosis, foam cell, efferocytosis and phagocytosis assays

Foam cell assays were performed by incubating cultured cells with DiO-Ac-LDL (Bioquote). Apoptosis assays were performed by exposing cultured cells to UV light, then staining them for Annexin V-FITC and PI (Life Technologies). Phagocytosis assays were performed by incubating cultured cells with Fluoresbrite Yellow-Green microspheres (Polysciences Inc.). Efferocytosis assays were performed by incubating cultured cells with Calcein AM-labelled apoptotic Jurkat cells [4].

Transfection of murine GMCSF Macrophages with RNAi Oligos

Bone marrow macrophages were generated as above and were then plated at 1 x 10^6 in a 12 well plate with 20ng/ml fresh GMCSF overnight at 37^0C. The next day, the oligo (ON-TARGET plus SMART pool siRNA for mouse Mfge8, Itgb3 or a non-targeting control, Dharmacon), Dharmafect 1 (Dharmacon) and Opti-mem (Gibco) mix was prepared to give a final concentration of 100nM of oligo and left to incubate 20min at RT. The macrophage growth media was then replaced with the relevant oligo-mix (in serum-free RPMI) and incubated at 37^0C for 2hrs. After removal of the transfection complexes, 1ml of RPMI with 5%FCS was added to the well and the plate incubated for 48hrs at 37^oC. The efferocytosis assay was then performed as described.

Measurement of Mfge8 release

Supernatants from GMCSF-derived bone marrow cultures were removed after one week and stored at -80^oC for batch analysis. A commercially available mouse MFGE8 ELISA kit was used (Biolegend) in accordance with the manufacturer's instructions. Samples were run in duplicate.

Flow cytometric analysis

Aortas and PALNs were harvested from 20 week old $ApoE^{-/-}$ and $ApoE^{-/-}Irf5^{-/-}$ mice. Aortas, including the aortic arch, thoracic and abdominal portions were incubated with an enzyme cocktail containing 230U/ml collagenase I, 144U/ml collagenase XI, 64U/ml hyalurodinase, 61U/ml DNase, 2mM CaCl2 and 1mg/ml soybean trypsin inhibitor (Sigma-Aldrich) in PBS 1% FCS for 1 hour at 37°C. Post-digestion, single cell suspensions were obtained by mashing aortas through a 70µm cell strainer. Enzymatic digestion was not required for the PALN. For macrophage cell cultures, both adherent and non-adherent GMCSF matured macrophages were collected. All cell types were then incubated with 0.5mg/ml of Mouse Fc block (BD Biosciences) for 10 min at 4°C before incubation with antibodies.

Antibodies against the following antigens were used: MHCII (clone M5/114, BD Pharmingen), F4/80 (clone BM8, Biolegend), CD11c (clone N418, Biolegend), CD11b (clone M1/70 Biolegend), CD103 (clone 2E7, Biolegend), MerTK (clone 108928, R&D systems), Itgb3 (clone 2C9.G2, BD Biosciences) and CD45 (clone 30-F11, Biolegend). Cells were additionally stained with the Live/Dead Fixable Dead Cell Stain kit according to the manufacturer's instructions (Life technologies). Cells were washed and then fixed with BD CellFIX (BD Biosciences). All gates were based on fluorescence minus one (FMO) controls. Fluorescent labels were detected with a BD LSRII flow cytometer (BD Biosciences) and results were analyzed using FlowJo software 10.07 (FlowJo, USA). The main myeloid cell populations were gated as previously described by Helft *et al* [5].

Real time quantitative PCR

Total RNA was extracted from GMCSF matured macrophages, aorta and PALN from 20 week old $ApoE^{-/-}$ and $ApoE^{-/-}Irf5^{-/-}$ mice using a Qiagen RNeasy mini kit, according to manufacturer's instructions. Total RNA was reverse transcribed to cDNA using a High Capacity Reverse Transcription Kit (Life Technologies). Following preamplification (14 cycles), RT-PCR was performed using either custom TaqMan Array microfluidic cards or individual TaqMan Gene Expression Assays and TaqMan universal PCR Master Mix (Life technologies) on an ABI 7900HT fast real-time PCR system (Applied Biosystems). PCR amplification was carried out for 40 cycles. All samples were analysed in triplicate and were normalized to 18s. The 2-ΔΔCt method was used to analyse the relative changes in gene expression.

Chromatin immunoprecipitation and next-generation sequencing

The IRF5 ChIP-seq analysis were performed as previously described (Accession number: E-MTAB-2661)[6]. 300 million GMCSF matured bone marrow derived macrophages from wild type and $Irf5^{-/-}$ mice were used for IRF5 ChIP following stimulation with LPS for 0 and 2hrs in duplicate. Cells were fixed for 10 minutes with 1% formaldehyde, quenched with 125mM of Tris pH7.5 and washed with ice-cold PBS. Nuclear lysates were isolated as previously described and sonicated with a Bioruptor (Diagenode) to obtain chromatin fragment sizes that average 300bp. Each lysate was immunoprecipitated with 10µg of IRF5 antibody (Abcam; ab21689). ChIP was performed as described previously. ChIPped DNA was quantified with the Quant-iT dsDNA High Sensitivity Assay Kit (Invitrogen #Q33120). DNA yields ranged from 10–20 ng. The ChIP-Seq datasets were generated using 50bp paired end sequencing. Reads were trimmed using Trimmomatic, and mapped to the mm10 genome using Bowtie2. Before peak calling with MACS2 using the following settings: --mfold 10 30 --gsize mm --qvalue 0.05. Called peaks in the WT samples were then filtered against those in $Irf5^{-/-}$ to exclude any false positives. Read coverage profiles were computed with 25bp resolution and normalized by reads per kilobase per million mapped reads (RPKM) using deepTools. Called peaks and read coverage profiles were visualized using IGV.

Human carotid plaque sample preparation and histology

Human carotid plaques from the Carotid Plaque Imaging Project (CPIP) biobank (Malmö, Sweden) were analyzed. Plaques were collected at carotid endarterectomy and the indications for surgery were plaques associated with ipsilateral symptoms (Transitory ischemic attack, stroke or amaurosis fugax) and >70% stenosis, measured by duplex, or plaques not associated with symptoms and stenosis >80%.

After surgical removal, plaques were snap-frozen in liquid nitrogen. Fragments (1mm) from the most stenotic region of the plaque were used for histology. Sections were stained with antibodies against CD68 (Dako cytomation, 1.85µg/mL), alpha-smooth muscle actin (Dako cytomation, 0.71µg/mL), IRF5 (Abcam, 20µg/mL) or CD11c (Abcam, 1µg/mL) or relevant isotype control antibodies. Following incubation with an appropriate secondary antibody or MACH3 mouse polymerase kit (Biocare Medical), 3,3'- diaminobenzidine (DAB) was used for staining detection and nuclei were counterstained with Mayer´s hematoxylin. Quantification of the area of plaque

staining positive (% area) for the different stains was performed blind using BiopixiQ 2.1.8 (Gothenburg, Sweden) after imaging with ScanScope Console Version 8.2 (LRI imaging AB, Vista CA, USA).

Supplemental Tables

Supplemental Table 1: Summary of results of robust 2-way ANOVA analysis of the data shown in Figure 1

Variable	Factor	p-value Area (%)	p-value Area (absolute)
Lesion area	Genotype	0.029	0.129
	Time (weeks)	0.001	0.001
	Genotype:Time	0.749	0.273
Necrotic core	Genotype	0.001	0.001
	Time (weeks)	0.001	0.001
	Genotype:Time	0.001	0.001

Supplemental Table 2: Summary of results of robust 2-way ANOVA analysis of the data shown in Figure 2

Variable	Factor	p-value Area (%)	p-value Area (absolute)
CD68	Genotype	0.045	0.278
	Time (weeks)	0.120	0.001
	Genotype:Time	0.860	0.750
CD11c	Genotype	0.001	0.001
	Time (weeks)	0.216	0.003
	Genotype:Time	0.015	0.013
CD206	Genotype	0.007	0.009
	Time (weeks)	0.051	0.001
	Genotype:Time	0.435	0.174

Supplemental Table 3: Summary of results of robust mixed 2-way ANOVA analysis of the data shown in Figure 4

Variable	Factor	p-value Area (%)	p-value Area (absolute)
Necrotic core	Genotype	0.00496	0.01165
	Stress	0.02844	0.03309
	Genotype:Stress	0.00153	0.00263
CD68	Genotype	0.757	0.982
	Stress	0.122	0.394
	Genotype:Stress	0.774	0.207
CD11c	Genotype	0.00427	0.0264
	Stress	0.17554	0.0962
	Genotype:Stress	0.17594	0.0934
Asma	Genotype	0.0348	0.068
	Stress	0.0134	6.66e-06
	Genotype:Stress	0.1129	2.36e-03

Supplemental Table 4: Summary of results of robust 2-way ANOVA analysis of the data shown in Supplemental Figure 2

Variable	Factor	p-value Area (%)	p-value Area (absolute)
Asma	Genotype	0.129	0.938
	Time (weeks)	0.047	0.003
	Genotype:Time	0.116	0.983
HO-1	Genotype	0.001	0.336
	Time (weeks)	0.817	0.001
	Genotype:Time	0.003	0.221
iNOS	Genotype	0.284	0.315
	Time (weeks)	0.001	0.001
	Genotype:Time	0.365	0.315

Supplemental Table 5: Summary of results of robust mixed 2-way ANOVA analysis of the data shown in Supplemental Figure 3

Variable	Factor	p-value
IMR	Genotype	0.485
	Stress	0.140
	Genotype:Stress	0.298

Supplemental Table 6. Final body weight and serum cholesterol levels in ApoE$^{-/-}$ and ApoE$^{-/-}$Irf5$^{-/-}$ mice

Mouse genotype	Age (wk)	Final body weight ± SEM (g)	p-value vs. ApoE$^{-/-}$	Serum cholesterol ± SEM (mg/dL)	p-value vs. ApoE$^{-/-}$
ApoE$^{-/-}$	15	30.2 ± 0.8	-	286.2 ± 30.9	-
ApoE$^{-/-}$Irf5$^{-/-}$	15	32.5 ± 0.5	**0.019**	301.6 ± 15.5	0.620
ApoE$^{-/-}$	20	32.1 ± 0.5	-	382.8 ± 58	-
ApoE$^{-/-}$Irf5$^{-/-}$	20	32.1 ± 0.5	0.991	510.4 ± 81.2	0.213
ApoE$^{-/-}$	27	32.9 ± 0.6	-	471.8 ± 88.9	-
ApoE$^{-/-}$Irf5$^{-/-}$	27	33.6 ± 0.6	0.417	467.9 ± 69.6	0.954

Supplemental Table 7. Body weight and final serum cholesterol levels in ApoE$^{-/-}$ and ApoE$^{-/-}$Irf5$^{-/-}$ mice that underwent cast-placement surgery

Mouse genotype	Age (wk)	Final body weight ± SEM (g)	p-value vs. ApoE$^{-/-}$	Serum cholesterol ± SEM (mg/dL)	p-value vs. ApoE$^{-/-}$
ApoE$^{-/-}$	15-17	31.0 ± 0.4	-	-	-
ApoE$^{-/-}$Irf5$^{-/-}$	15-17	34.1 ± 0.5	**<0.0001**	-	-
ApoE$^{-/-}$	24-26	32.7 ± 0.7	-	564.6 ± 77.3	-
ApoE$^{-/-}$Irf5$^{-/-}$	24-26	40.4 ± 1.7	**0.0008**	618.7 ± 58	0.5914

Supplemental Table 8. iNOS, CD206 and HO-1 expression in perivascular shear stress altering cast-induced lesions in ApoE$^{-/-}$ and ApoE$^{-/-}$Irf5$^{-/-}$ mice

Mouse Genotype	Flow region	iNOS expression ± SEM (%)	p-value vs. ApoE$^{-/-}$	CD206 expression ± SEM (%)	p-value vs. ApoE$^{-/-}$	HO-1 expression ± SEM (%)	p-value vs. ApoE$^{-/-}$
ApoE$^{-/-}$	Low	15.7±2.7	-	4.0±0.9	-	8.8±4.4	-
ApoE$^{-/-}$Irf5$^{-/-}$	Low	13.6±5.0	0.76	6.3±3.1	0.73	3.0±1.8	0.26
ApoE$^{-/-}$	Oscillatory	14.5±3.1	-	10.2±1.2	-	11.3±5.2	-
ApoE$^{-/-}$Irf5$^{-/-}$	Oscillatory	9.7±3.4	0.35	11.0±2.8	0.91	11.4±2.8	0.61

Supplemental Table 9. Percentage of foam cells in bone marrow derived cultures of ApoE$^{-/-}$ and ApoE$^{-/-}$Irf5$^{-/-}$ mice

Sorted population	Treatment	ApoE$^{-/-}$ Mean ± SEM (%)	ApoE$^{-/-}$Irf5$^{-/-}$ Mean ± SEM (%)	p-value vs. ApoE$^{-/-}$
CD11c+	None	31.1 ± 7.5	30.6 ± 7.3	0.965
	LPS	29.1 ± 10.5	30.2 ± 7.3	0.939
CD11c-	None	27.2 ± 1.7	28.0 ± 1.8	0.768
	LPS	24.1 ± 4.5	25.3 ± 4.1	0.854

Supplemental Table 10. Percentage of phagocytosis in bone marrow-derived cultures of ApoE$^{-/-}$ and ApoE$^{-/-}$Irf5$^{-/-}$ mice

Sorted population	Treatment	ApoE$^{-/-}$ Mean ± SEM (%)	ApoE$^{-/-}$Irf5$^{-/-}$ Mean ± SEM (%)	p-value vs. ApoE$^{-/-}$
CD11c+	None	33.9 ± 5.8	27.9 ± 2.4	0.374
	LPS	36.5 ± 5.7	30.6 ± 3.0	0.394
CD11c-	None	27.7 ± 3.0	32.7 ± 4.4	0.380
	LPS	33.1 ± 5.2	32.4 ± 3.5	0.915

Supplemental Table 11. Percentage of apoptotic cells in bone marrow GMCSF-derived cultures of ApoE$^{-/-}$ and ApoE$^{-/-}$Irf5$^{-/-}$ mice

Sorted population	Treatment	Cell population	ApoE$^{-/-}$ Mean ± SEM (%)	ApoE$^{-/-}$Irf5$^{-/-}$ Mean ± SEM (%)	p-value vs. ApoE$^{-/-}$
CD11c+	None	Live	20.5 ± 10.6	20.1 ± 10.6	0.984
		Annexin +	59.2 ± 17.1	67.0 ± 13.7	0.734
		Annexin+ PI+	10.4 ± 1.6	6.9 ± 1.9	0.211
	LPS	Live	23.5 ± 11.6	20.6 ± 9.7	0.856
		Annexin +	49.6 ± 14.3	65.1 ± 14.1	0.471
		Annexin+ PI+	11.2 ± 5.7	7.8 ± 1.8	0.169
CD11c-	None	Live	27.8 ± 12.4	21.6 ± 9.8	0.709
		Annexin +	59.9 ± 11.9	65.3 ± 14.7	0.784
		Annexin+ PI+	8.9 ± 6.1	6.1 ± 1.9	0.410
	LPS	Live	27.5 ± 12.3	24.9 ± 10.6	0.879
		Annexin +	56.5 ± 16.2	66.1 ± 13.0	0.660
		Annexin+PI+	14.9 ± 3.6	3.1 ± 0.8	**0.00005**

Supplemental Figures

Supplemental Figure 1

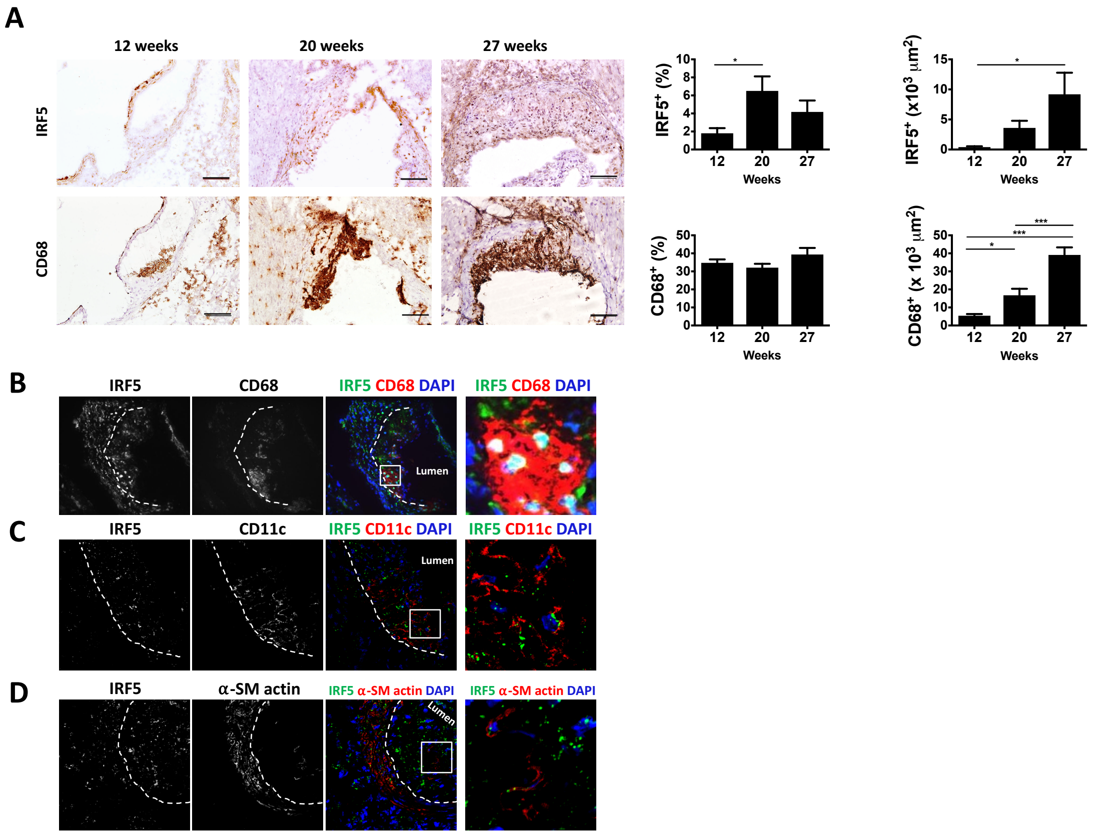

Supplemental Figure 1. IRF5 expression in aortic root lesions of ApoE$^{-/-}$ mice. **A)** Representative images of aortic root sections from ApoE$^{-/-}$ mice aged 12, 20 and 27 weeks stained with an antibody against IRF5 or CD68 (brown staining) and hematoxylin. Scale bar = 100µm. Graphs show aortic root lesion area staining positive (x10^3 µm^2 and %) for CD68 and IRF5 in 12 to 27 week old ApoE$^{-/-}$ mice. Bars show mean + SEM. n=3-4 *p<0.05, ***p<0.001 **B-D)** Dual immunofluorescent staining and confocal analysis of IRF5 and CD68 (**B**), CD11c **(C)** or αSM actin **(D)** expression in aortic root sections of 30 week old ApoE-/- mice fed a chow diet. Aortic root sections were dual immunostained with an antibody against IRF5 (Alexa 488, green emission), with tyramide signal amplification, and with either an antibody against the pan-macrophage marker CD68 (Alexa 568, red emission), an antibody against CD11c (Alexa 568, red emission) or an antibody against αSM actin (Cy3, red emission). Sections were counterstained with DAPI. Composite pseudocolour images; Green, Irf5; Red, CD68, CD11c or αSM actin and Blue, DAPI nuclear dye. Dotted line denotes internal elastic lamina. Zoom image of area denoted by white box in composite pseudocolour image.

Supplemental Figure 2

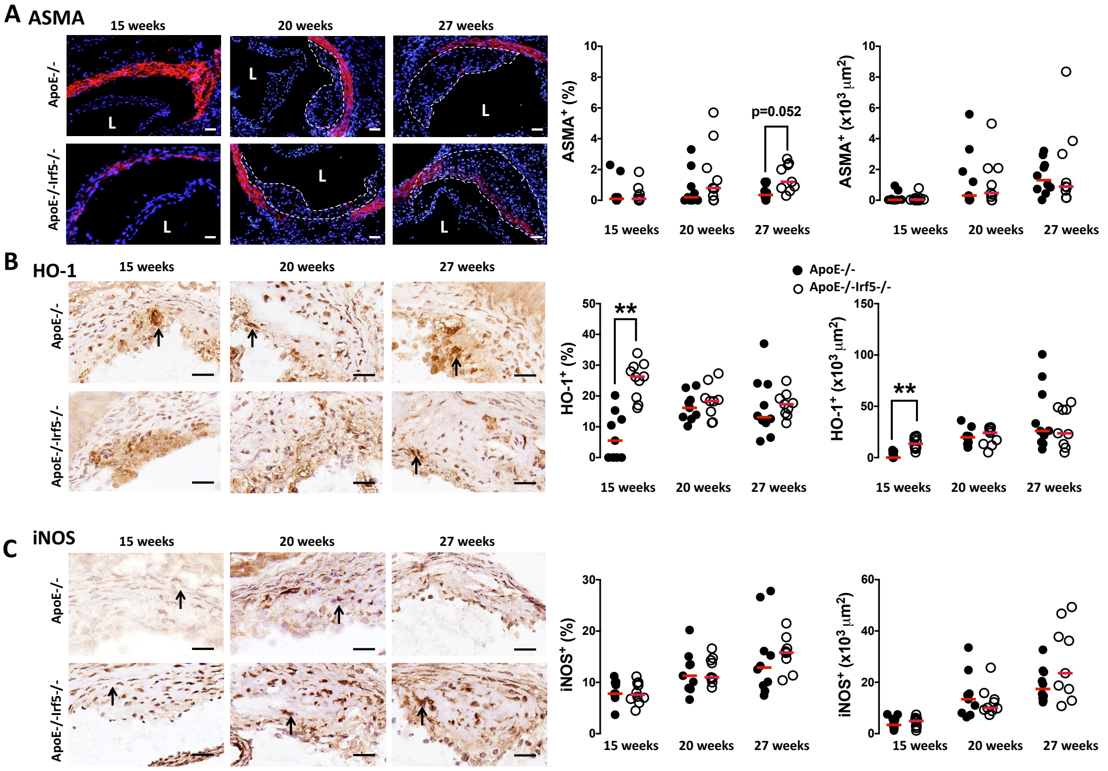

Supplemental Figure 2. Immunohistochemistry in aortic root lesions of ApoE$^{-/-}$ versus ApoE$^{-/-}$Irf5$^{-/-}$ mice. A) Representative photomicrographs of aortic root sections from 15, 20 and 27 week old ApoE$^{-/-}$ and ApoE$^{-/-}$Irf5$^{-/-}$ mice stained with an antibody against smooth muscle cell α-actin (ASMA) (Cy3-red) and DAPI (blue). L = lumen. Graphs show aortic root lesion area staining positive (x10^3 µm^2 and %) for ASMA. Scale bars = 100µm. **B)** Representative photomicrographs of aortic root sections from 15, 20 and 27 week old ApoE$^{-/-}$ and ApoE$^{-/-}$Irf5$^{-/-}$ mice stained with an antibody against HO-1 (brown staining) and hematoxylin. Graphs show aortic root lesion area staining positive (x10^3 µm^2 and %) for HO-1. **C)** Representative photomicrographs of aortic root sections from 15, 20 and 27 week old ApoE-/- and ApoE-/-Irf5-/- mice stained with an antibody against iNOS (brown staining) and hematoxylin. Graphs show aortic root lesion area staining positive (x10^3 µm^2 and %) for iNOS. Arrows highlight positive cells. Each circle represents the mean positive area per individual mouse. Horizontal line denotes group mean. **p<0.01, n=9-10.

Supplemental Figure 3

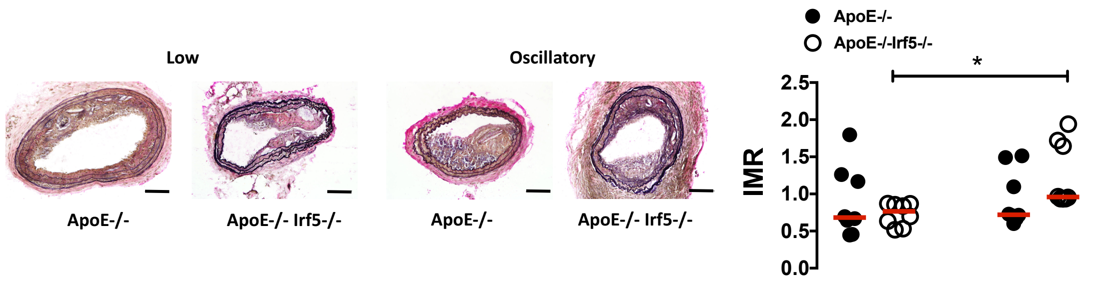

Supplemental Figure 3. Lesion size in a murine model of TCFA. ApoE$^{-/-}$ (black circles) and ApoE$^{-/-}$Irf5$^{-/-}$ mice (white circles) were placed on high fat diet at 17-18 weeks of age. After 2 weeks, a perivascular shear stress altering cast was surgically placed around the common carotid artery and left in place for 9 weeks. Representative images of carotid artery sections stained with Elastin-Van Gieson stain, Scale bars = 100µm. Each circle represents the mean Intima:Media ratio (IMR) of carotid artery regions 9 weeks after cast placement per individual mouse. Horizontal line denotes group median. *$p<0.05$ n=8

Supplemental Figure 4

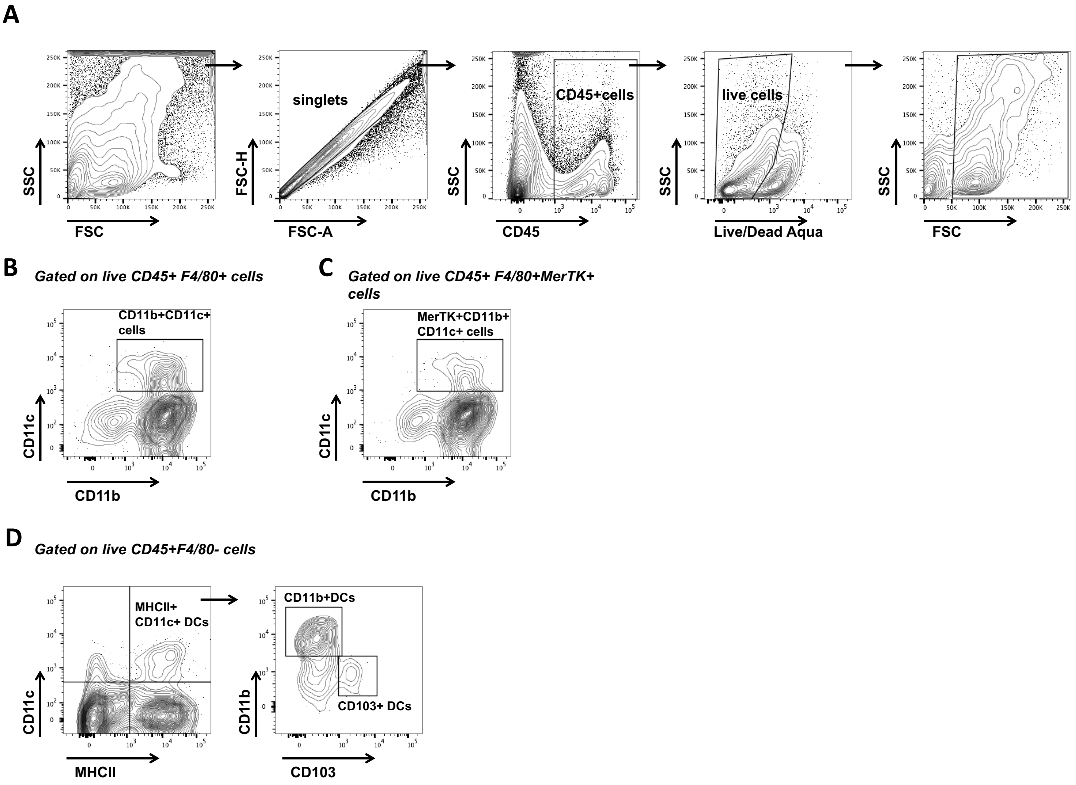

Supplemental Figure 4. Representative staining and gating of aortic myeloid cell subsets. Aortas were harvested and enzymatically digested then stained with antibodies against myeloid cell markers and a live/dead stain. **A)** Representative plots showing cell gating. To gate myeloid cells, doublets were excluded before CD45+ were gated. Live CD45+ cells were then gated. **B)** Representative plot showing macrophage CD11b+CD11c+ gate (cells gated as CD45+F4/80+) **C)** Representative plot showing macrophage CD11b+CD11c+ gate (cells gated as CD45+F4/80+MerTK+) **D)** Representative plots showing dendritic cell gating. CD45+F4/80- cells were further gated as CD11c+MHCII+ and these cells were then separated on the basis of CD11b and CD103 expression.

Supplemental Figure 5

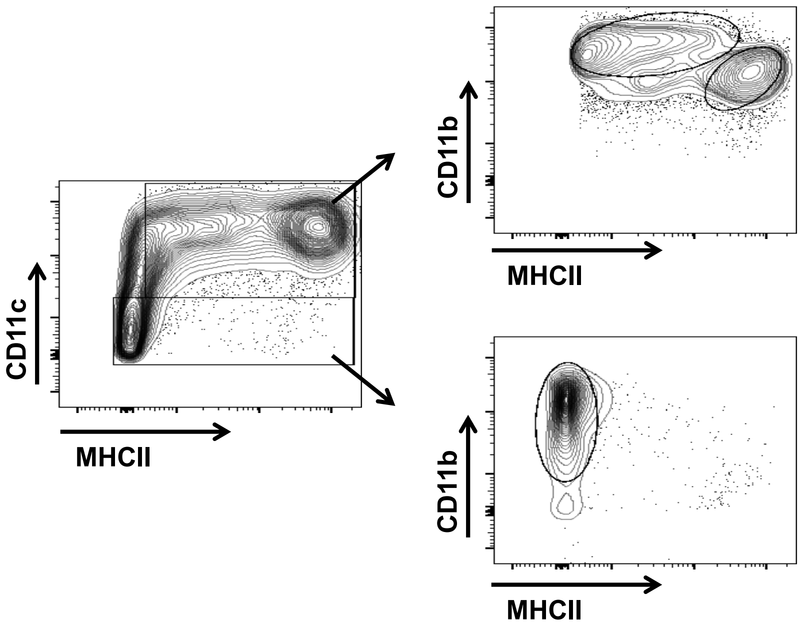

Supplemental Figure 5. Representative staining and gating of bone marrow GMCSF cultured myeloid cell subsets. Cells were gated as MHCII$^{int-high}$ and CD11c+ or MHCII^{low}CD11c-. CD11c+MHCII$^{int-high}$ cells were then further gated as CD11b^{high}MHCII^{int} to identify macrophages and CD11b^{int}MHCII^{high} to identify dendritic cells. For CD11c- cells, cells were further gated as CD11b$^{low-high}$ and MHCII$^{low/int}$.

Supplemental Figure 6

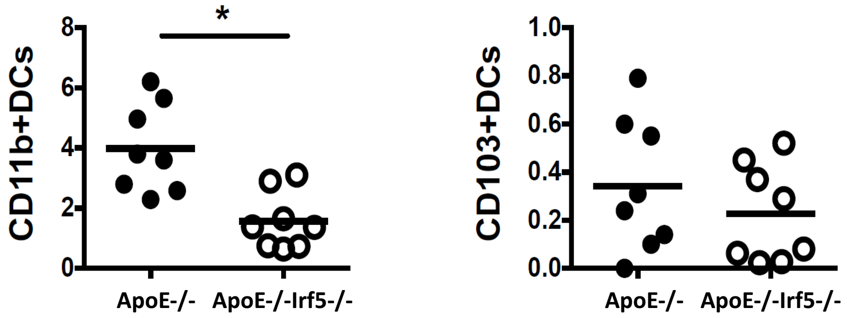

Supplemental Figure 6. CD11b+ dendritic cells are decreased in the aorta of IRF5 deficient mice. Aortas were harvested from 21-24 week old ApoE$^{-/-}$ and ApoE$^{-/-}$ Irf5$^{-/-}$ mice. Single cell suspensions were then stained with antibodies against myeloid cell markers and analysed by flow cytometry. Dead cells and debris were excluded from the analysis and cells were gated on CD45+ cells. Graphs show the numbers of aortic CD45+F4/80-CD11c+MHCII+ cells that were CD11b+CD103- (left graph) or CD11b-CD103+ (right graph). Each circle represents an individual mouse. Horizontal line denotes group mean. *p<0.05 n=8.

Supplemental Figure 7

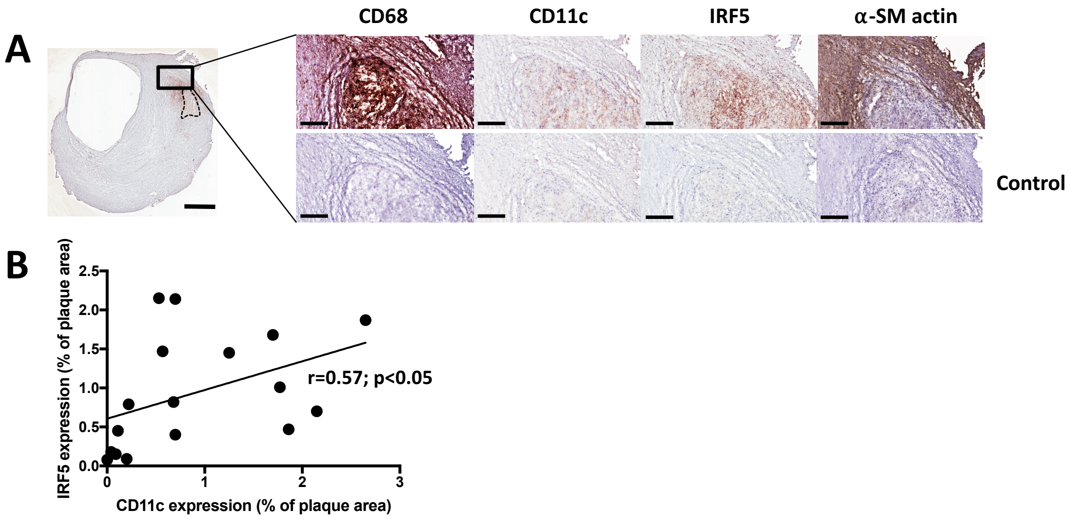

Supplemental Figure 7. IRF5 is expressed by CD11c+ cells in human carotid plaques. A) Representative images of a human carotid plaque stained with antibodies against CD68, CD11c, IRF5 and αSM actin (brown staining) and hematoxylin. Dashed line denotes necrotic core. Far left image: x2 magnification, scale bar = 1mm. Other images represent x10 magnification of area of plaque denoted by box in x2 magnification image, scale bar = 200μm. **B)** Scatter plot graph showing the Spearman correlation between IRF5 and CD11c positively stained areas in the human carotid plaques. Each dot represents an individual human carotid plaque r=0.57, p<0.05; n=17.

Supplemental Figure 8

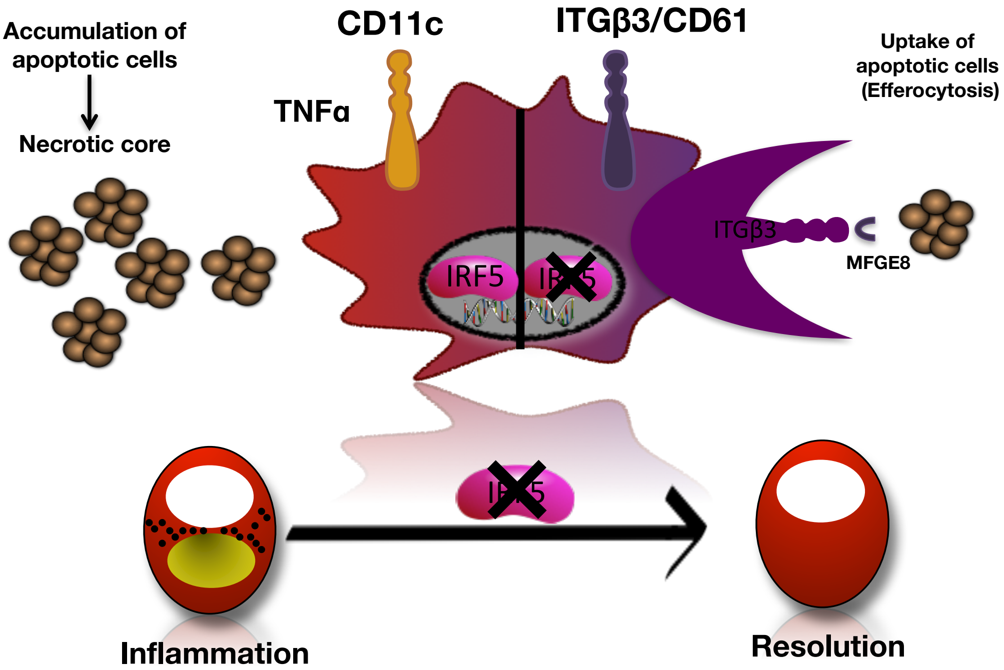

Supplemental Figure 8. IRF5 controls the formation of the necrotic core impairing efferocytosis. The diagram summarizes the findings of our study. IRF5 drives the expression of CD11c on activated macrophages with pro-inflammatory properties. Its deletion in atherosclerosis causes a switch from CD11c expressing to ITGB3 expressing macrophages enhancing efferocytosis and preventing the formation of the necrotic core.

Supplemental References

1. Cheng C, Tempel D, van Haperen R, van Der Baan A, Grosveld F, Daemen MJAP, Krams R, de Crom R. Atherosclerotic lesion size and vulnerability are determined by patterns of fluid shear stress. *Circulation*. 2006;113:2744-2753. doi:10.1161/CIRCULATIONAHA.105.590018
2. Cole JE, Navin TJ, Cross AJ, Goddard ME, Alexopoulou L, Mitra AT, Davies AH, Flavell RA, Feldmann M, Monaco C. Unexpected protective role for Toll-like receptor 3 in the arterial wall. *Proc Natl Acad Sci U S A*. 2011;108:2372-2377. doi:10.1073/pnas.1018515108
3. Thorp E, Cui D, Schrijvers DM, Kuriakose G, Tabas I. Mertk receptor mutation reduces efferocytosis efficiency and promotes apoptotic cell accumulation and plaque necrosis in atherosclerotic lesions of apoe-/- mice. *Arterioscler Thromb Vasc Biol*. 2008;28:1421-1428. doi:10.1161/ATVBAHA.108.167197
4. Seimon TA, Wang Y, Han S, Senokuchi T, Schrijvers DM, Kuriakose G, Tall AR, Tabas IA. Macrophage deficiency of p38alpha MAPK promotes apoptosis and plaque necrosis in advanced atherosclerotic lesions in mice. *J Clin Invest* 2009;119:886-898. doi:10.1172/JCI37262
5. Helft J, Bottcher J, Chakravarty P, Zelenay S, Huotari J, Schraml BU, Goubau D, Reis e Sousa C. GM-CSF Mouse Bone Marrow Cultures Comprise a Heterogeneous Population of CD11c(+)MHCII(+) Macrophages and Dendritic Cells. *Immunity*. 2015;42:1197-1211. doi:10.1016/j.immuni.2015.05.018
6. Saliba DG, Heger A, Eames HL, Oikonomopoulos S, Teixeira A, Blazek K, Androulidaki A, Wong D, Goh FG, Weiss M, Byrne A, Pasparakis M, Ragoussis J, Udalova IA. IRF5:RelA interaction targets inflammatory genes in macrophages. *Cell Rep*. 2014;8:1308-1317. doi:10.1016/j.celrep.2014.07.034
